# Supplementary material for: Investigation of gate dielectric interface on contact resistance of short channel organic thin film transistors (OTFT)
Source: PLoS One. 2025 Sep 11;20(9):e0326929. doi: 10.1371/journal.pone.0326929 (PMC12425321; doi:10.1371/journal.pone.0326929)
Supplement: Attachment — Submitted filename: PONE-D-24-59959.pdf [file pone.0326929.s001.pdf]

PLOS ONE

Investigation of Gate Dielectric Interface on Contact Resistance of Short Channel Organic Thin Film Transistors (OTFT)

--Manuscript Draft--

**Manuscript Number:**	PONE-D-24-59959
**Article Type:**	Research Article
**Full Title:**	Investigation of Gate Dielectric Interface on Contact Resistance of Short Channel Organic Thin Film Transistors (OTFT)
**Short Title:**	A Study of Short Channel Organic Thin Film Transistors' Contact Resistance and Gate Dielectric Interface
**Corresponding Author:**	Durga Prakash Matta, Ph.D.<br>SRM University AP - Amaravati: SRM University AP<br>Guntur, Armed Forces Pacific INDIA
**Keywords:**	Contact resistance; Hybrid dielectric; Leakage Current density; Organic thin film transistor (OTFT); Interface charge density
**Abstract:**	This study provides a comprehensive analysis of the impact of the interfacial properties on the performance of organic thin-film transistors (OTFTs) with a hybrid dielectric of Al2O3/PVP compared to single-layer dielectrics of Al2O3 and PVP. The analyses were performed using the 2D Silvaco Atlas numerical simulator, which conducted a detailed numerical investigation into how varying the thickness ratio of Al2O3 and PVP in the dielectric affects contact resistance and off-state current in short-channel OTFTs. High-K dielectric materials, such as Al2O3, offer low threshold voltages but lead to increased contact resistance and leakage current, while low-K dielectrics like PVP reduce leakage current but suffer from lower mobility and higher contact resistance. By utilizing a hybrid Al2O3/PVP dielectric, we successfully reduced the contact resistance to 4.84 KΩ, as extracted from VDS-ID characteristics at a gate voltage of -2V. Additionally, contact resistance significantly influenced the off-state current, particularly in devices of short channel length (1 μm). The PVP layer, with thicknesses ranging from 2.4 nm to 4.2 nm, effectively reduced charge carrier traps at the semiconductor/dielectric interface, enhancing mobility. Furthermore, hysteresis effects were examined through C-V characteristics by sweeping the gate voltage from -3V to +3V. These findings highlight the trade-offs in optimizing PVP thickness to balance interface quality and electrical performance in hybrid dielectric OTFTs.
**Order of Authors:**	Lingala Prasanthi
	Asisa Kumar Panigrahy, PhD
	Subhashini Tata
	Rohith Bala Jaswanth B
	Talla Srinivasa Rao
	Durga Prakash Matta, Ph.D.
**Additional Information:**	
**Question**	**Response**
**Financial Disclosure**<br><br>Enter a financial disclosure statement that describes the sources of funding for the work included in this submission. Review the submission guidelines for detailed requirements. View published research articles from *PLOS ONE* for specific examples.	The author(s) received no specific funding for this work.

Powered by Editorial Manager® and ProduXion Manager® from Aries Systems Corporation

This statement is required for submission and **will appear in the published article** if the submission is accepted. Please make sure it is accurate.<br><br>**Funded studies**<br>Enter a statement with the following details:<br>• Initials of the authors who received each award<br>• Grant numbers awarded to each author<br>• The full name of each funder<br>• URL of each funder website<br>• Did the sponsors or funders play any role in the study design, data collection and analysis, decision to publish, or preparation of the manuscript?<br><br>Did you receive funding for this work?	
**Competing Interests**<br><br>Use the instructions below to enter a competing interest statement for this submission. On behalf of all authors, disclose any competing interests that could be perceived to bias this work—acknowledging all financial support and any other relevant financial or non-financial competing interests.<br><br>This statement is required for submission and **will appear in the published article** if the submission is accepted. Please make sure it is accurate and that any funding sources listed in your Funding Information later in the submission form are also declared in your Financial Disclosure statement.<br><br>View published research articles from *PLOS ONE* for specific examples.	The authors have declared that no competing interests exist.

Powered by Editorial Manager® and ProduXion Manager® from Aries Systems Corporation

**NO authors have competing interests**<br><br>Enter: *The authors have declared that no competing interests exist.*<br><br>**Authors with competing interests**<br><br>Enter competing interest details beginning with this statement:<br><br>*I have read the journal's policy and the authors of this manuscript have the following competing interests: [insert competing interests here]*<br><br>* typeset	
**Ethics Statement**<br><br>Enter an ethics statement for this submission. This statement is required if the study involved:<br><br>• Human participants<br>• Human specimens or tissue<br>• Vertebrate animals or cephalopods<br>• Vertebrate embryos or tissues<br>• Field research<br><br>Write "N/A" if the submission does not require an ethics statement.<br><br>General guidance is provided below. Consult the submission guidelines for detailed instructions. **Make sure that all information entered here is included in the Methods section of the manuscript.**	N/A

**Format for specific study types**<br><br>**Human Subject Research (involving human participants and/or tissue)**<br>• Give the name of the institutional review board or ethics committee that approved the study<br>• Include the approval number and/or a statement indicating approval of this research<br>• Indicate the form of consent obtained (written/oral) or the reason that consent was not obtained (e.g. the data were analyzed anonymously)<br><br>**Animal Research (involving vertebrate animals, embryos or tissues)**<br>• Provide the name of the Institutional Animal Care and Use Committee (IACUC) or other relevant ethics board that reviewed the study protocol, and indicate whether they approved this research or granted a formal waiver of ethical approval<br>• Include an approval number if one was obtained<br>• If the study involved *non-human primates*, add *additional details* about animal welfare and steps taken to ameliorate suffering<br>• If anesthesia, euthanasia, or any kind of animal sacrifice is part of the study, include briefly which substances and/or methods were applied<br><br>**Field Research**<br><br>Include the following details if this study involves the collection of plant, animal, or other materials from a natural setting:<br>• Field permit number<br>• Name of the institution or relevant body that granted permission	
**Data Availability**<br><br>Authors are required to make all data underlying the findings described fully available, without restriction, and from the time of publication. PLOS allows rare exceptions to address legal and ethical concerns. See the PLOS Data Policy and FAQ for detailed information.	Yes - all data are fully available without restriction

A Data Availability Statement describing where the data can be found is required at submission. Your answers to this question constitute the Data Availability Statement and **will be published in the article**, if accepted.<br><br>**Important:** Stating 'data available on request from the author' is not sufficient. If your data are only available upon request, select 'No' for the first question and explain your exceptional situation in the text box.<br><br>Do the authors confirm that all data underlying the findings described in their manuscript are fully available without restriction?	
**Describe where the data may be found in full sentences. If you are copying our sample text, replace any instances of XXX with the appropriate details.**<br><br>• If the data are **held or will be held in a public repository**, include URLs, accession numbers or DOIs. If this information will only be available after acceptance, indicate this by ticking the box below. For example: *All XXX files are available from the XXX database (accession number(s) XXX, XXX.*).<br>• If the data are all contained **within the manuscript and/or Supporting Information files**, enter the following: *All relevant data are within the manuscript and its Supporting Information files.*<br>• If neither of these applies but you are able to provide **details of access elsewhere**, with or without limitations, please do so. For example:<br><br>*Data cannot be shared publicly because of [XXX]. Data are available from the XXX Institutional Data Access / Ethics Committee (contact via XXX) for researchers who meet the criteria for access to confidential data.*<br><br>*The data underlying the results presented in the study are available from (include the name of the third party*	All relevant data are within the manuscript

Powered by Editorial Manager® and ProduXion Manager® from Aries Systems Corporation

and contact information or URL).<br>• This text is appropriate if the data are owned by a third party and authors do not have permission to share the data.<br><br>* typeset	
Additional data availability information:	

Manuscript

Click here to access/download;Manuscript;Plos One_format_dielectric interface.docx

Investigation of Gate Dielectric Interface on Contact Resistance of Short Channel Organic Thin Film Transistors (OTFT)

Lingala Prasanthi[1], Asisa Kumar Panigrahy[2,*], Subhashini Tata[3], Rohith Bala Jaswanth B[4], Talla Srinivasa Rao[5] and Matta DurgaPrakash[1*]

[1]Department of ECE, SRM University-AP, Mangalagiri, Andhra Pradesh, 522240, India
[2]Department of ECE, Faculty of Science and Technology (IcfaiTech), ICFAI Foundation for Higher Education Hyderabad-501203, Telangana, India
[3]Department of ECE, Seshadri Rao Gudlavalleru Engineering College, Gudlavalleru, Andhra Pradesh 521356, India
[4]Department of Internet of Things, Seshadri Rao Gudlavalleru Engineering College, Gudlavalleru, Andhra Pradesh 521356, India
[5]Department of ECE, Aditya University, Surampalem, Andhra Pradesh 533437, India

[*]Corresponding Email: asisa@ifheindia.org and durgaprakash.m@srmap.edu.in

Abstract

This study provides a comprehensive analysis of the impact of the interfacial properties on the performance of organic thin-film transistors (OTFTs) with a hybrid dielectric of Al_2O_3/PVP compared to single-layer dielectrics of Al_2O_3 and PVP. The analyses were performed using the 2D Silvaco Atlas numerical simulator, which conducted a detailed numerical investigation into how varying the thickness ratio of Al_2O_3 and PVP in the dielectric affects contact resistance and off-state current in short-channel OTFTs. High-K dielectric materials, such as Al_2O_3, offer low threshold voltages but lead to increased contact resistance and leakage current, while low-K dielectrics like PVP reduce leakage current but suffer from lower mobility and higher contact resistance. By utilizing a hybrid Al_2O_3/PVP dielectric, we successfully reduced the contact resistance to 4.84 KΩ, as extracted from V_{DS}-I_D characteristics at a gate voltage of -2V. Additionally, contact resistance significantly influenced the off-state current, particularly in devices of short channel length (1 µm). The PVP layer, with thicknesses ranging from 2.4 nm to 4.2 nm, effectively reduced charge carrier traps at the semiconductor/dielectric interface, enhancing mobility. Furthermore, hysteresis effects were examined through C-V characteristics by sweeping the gate voltage from -3V to +3V. These findings highlight the trade-offs in optimizing PVP thickness to balance interface quality and electrical performance in hybrid dielectric OTFTs.

Index Terms: Contact resistance, Hybrid dielectric, Leakage Current density, Organic thin film transistor (OTFT), Interface charge density.

Introduction

Over the last few decades, organic semiconductors have been critical to advancing organic thin-film transistor technology. These transistors demonstrate a significant advancement in the field of flexible and wearable electronics due to their distinctive characteristics, such as flexibility, low-temperature processing at fabrication, and being suitable to fabricate on plastic substrates [1]. They serve as controller circuits for numerous applications, including active-matrix organic light-emitting diodes (AMOLEDS) [2], RFID tags [3], gas and chemical sensors, biosensors [5], memory applications [4], large flat panel displays [6], and so on. Extensive studies have been carried out on different organic semiconductors and dielectric layers crucial for improving OTFT performance. To achieve higher switching speeds and operate at high frequencies, scaling down the channel length is required. However, this approach will be ineffective unless contact resistance is minimized, as it adversely affects the performance of short-channel OTFT devices. Consequently, extensive research has been conducted to minimize contact resistance through methods such as contact doping [7-8], using compatible contact materials, and employing self-assembled monolayer (SAM) techniques [9-10]. Despite these efforts, several factors affect contact resistance, including the electron affinity of organic semiconductors, interface traps at the dielectric/semiconductor junction, and interface dipoles. The interface between the gate dielectric and the semiconductor significantly influences the electrical performance of an OTFT, affecting parameters like threshold voltage, mobility, and charge carrier accumulation at low gate voltages [11-12].

A number of researchers have explored how high-K inorganic dielectrics like Al_2O_3[13], HfO_2[14], Ta_2O_5[15], ZrO_2[16], and TiO_2[17] might be able to improve the performance of OTFTs. High-K materials possess a high dielectric constant, so increasing the dielectric gate capacitance can accumulate more charge carriers in the dielectric-semiconductor interface when a low V_{GS} voltage is applied. Consequently, the operating voltage is reduced. The issue is that the majority of high-K materials possess a narrow band gap, resulting in a leakage current between semiconductors and dielectric interface. Senkevinch and Desa [18] stated that the chosen dielectric should have a high dielectric constant, a wide band gap, minimal interface traps, and great thermal stability. However, the high K dielectric constant causes a high surface energy, which lets organic semiconductors deposit unevenly on the dielectric surface. This compromises the interface's quality, leading to defects and an increase in interface trap density. These consequences include a decline in mobility and enhances the contact resistance [19], as well as the need a high temperature for processing the high-K materials.

In order to address this issue, organic dielectric is deposited over high-k materials. This renders the dielectric surface hydrophobic, making it suitable for depositing organic semiconductors, results an organic-organic interface with a noninteracting interface [20]. This enhances interface quality and device performance. Organic dielectrics like polyvinylphenol (PVP), polyvinyl alcohol (PVA), polyimide (PI), polymethylmethacrylate (PMMA), and others that have been proposed as gate dielectrics for organic electronics typically exhibit low dielectric constants, resulting in low capacitance, reduced mobility, high operating voltages, and hysteresis loss. A solution was proposed to utilize bilayer gate dielectrics made up of high-k metal-oxide films covered with low-k polymer films. This would combine the benefits of high-k oxide, which has a high capacitance and low operating voltages, with polymer dielectrics, which have better interfaces with organic semiconductors. Numerous researchers have developed the bilayer dielectric structure of OTFT in order to incorporate the benefits of low-K organic polymers to preserve a good interface quality with the

organic semiconductor and high-K dielectrics for high capacitance. Liwei Shang and Ming Liu et al. [20] fabricated an OTFT by combining ZrO_2 and PMMA as a bilayer dielectric with CuPu as the organic semiconductor. This design showed reduced leakage current compared to using only a single layer of ZrO_2. Sagarika Khound et al. [21] examined the electrical properties of a hybrid dielectric made of pentacene-based cross-linked polyvinyl phenol (cPVP) and lanthanum oxide (La_2O_3) using a solution processing approach.

Still, all these bilayer devices were operating at voltages higher than 15 V. Hence, it is essential to develop an OTFT that operates at low voltages and is appropriate for portable uses such as sensors and wearable electronics. Developing OTFTs that can operate effectively at low voltage and high switching speed without compromising electrical performance or operational stability is

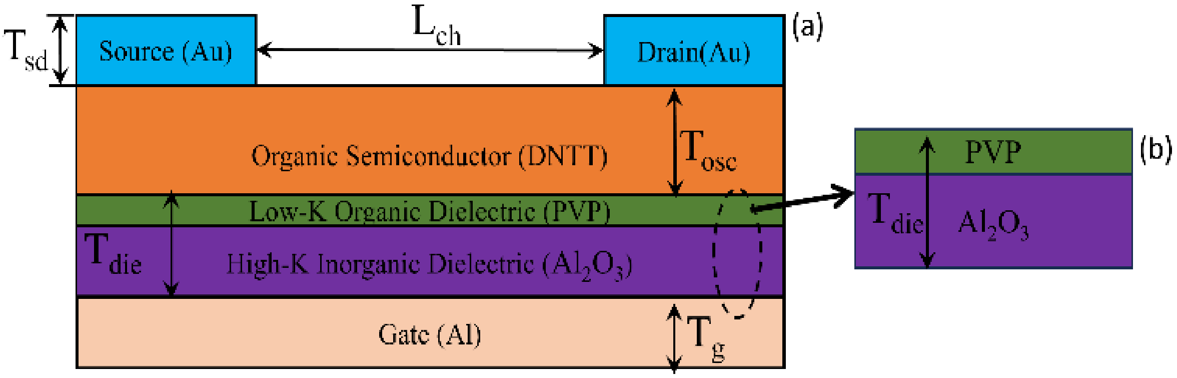

Fig 1. (a) A schematic representation of the simulated device structure for DNTT OTFTs (b) Varying dielectric layer single Al_2O_3, single PVP and hybrid layer with different thickness composition (P5A95, P20A80 and P40A60)

still a significant problem. On the other hand, investigations on contact resistance and leakage current in optimizing the hybrid dielectric layer have been rarely reported. There are only a few studies related to exploring the impact of dielectric on contact resistance [22-25]. Still, all these studies utilized single gate dielectric layers and therefore may not completely eliminate the influence of interface charge trap density and high surface energy at the interface.

This paper introduces a novel bottom gate OTFT based on dinaphtho[2,3 b:2',3'-f]thieno[3,2-b]thiophene (DNTT) as an organic semiconductor and employing a hybrid gate dielectric Al_2O_3/PVP, which minimizes the compromises between operating voltage and contact resistance in the short channel OTFTs as shown in Fig 1. Here, we explored the effect of single and hybrid dielectric layers of Al_2O_3 and PVP on the device parameters of the OTFT. We specifically investigated the impact of different interfacial effects on the electrical characterization of OTFTs, focusing on single dielectrics as well as various PVP thickness compositions within the hybrid dielectric layer. Although the devices are structurally identical, they differ in the composition of the PVP/Al_2O_3 hybrid dielectric layer. This variation allows us to understand the importance of optimizing PVP thickness in the hybrid dielectric composition and its impact on device performance. In this work, the simulated devices will be named according to the following nomenclature: "P##A##," where "##" refers to the ratio of thickness of the dielectric layer measured in percentage, "P" refers to the PVP layer, and "A" to the Al_2O_3 layer. Following this nomenclature, a device named P5A95 comprises a 5% PVP layer (0.6 nm) and a 95% Al_2O_3 layer (11.4 nm) out of 12 nm, P20A80, and P40A60. The P20A80 OTFT exhibited lower contact resistance compared to the other devices. It was observed that contact resistance affects both the off-state current and the mobility of the devices. We further examined hysteresis loss using CV characteristics by sweeping the gate voltage from -3V to +3V. The

P20A80 bilayer dielectric layer device operates at 3 V, features a high I_{ON}/I_{OFF} ratio of 1.83X10^{10}, a subthreshold swing of 57 mV/dec, low OFF- state current and negligible hysteresis loss.

Device Modelling and Simulation

Modelling of Parameters

The I_{DS}-V_{GS} characteristics of the OTFT are employed to extract the device parameters, which include mobility, transconductance, sub-threshold voltage, I_{ON}/I_{OFF} ratio, and threshold voltage. The I_{DS}-V_{GS} features are separated into two distinct regions: a linear region and a saturation region. Within the linear region, the accumulation of charge carriers occurs at the interface between the dielectric and semiconductor when V_{DS} is less than (V_{GS}-V_{TH}). In this case, the drain current of an OTFT based on DNTT can be determined in Equation (1) [26].

$$I_D = \frac{W}{L}\mu C_i(V_{GS}\text{-}V_{TH})V_{DS} \qquad (1)$$

Where W is width, L is length C_i represents oxide capacitance and μ is mobility. When V_{DS} exceeds (V_{GS}-V_{TH}) in the saturation area, I_{Dsat} is given by Equation (2)

$$I_{Dsat} = \frac{W}{2L}\mu C_i(V_{GS} - V_{TH})^2 \qquad (2)$$

$$\sqrt{I_{Dsat}} = \sqrt{K}(V_{GS} - V_{TH}) \qquad (3)$$

Equation (2) can be expressed as Equation (3), with $\sqrt{K}$ representing the slope and V_{TH} denoting the threshold voltage. To extract the threshold voltage of an organic thin-film transistor (OTFT), perform a linear fit on the x-intercept slope of the square root of the drain current $\sqrt{I_{Dsat}}$ versus the gate-source voltage V_{GS} plot. This linear fit can then be extrapolated on the x-axis to obtain the desired threshold voltage value. Transconductance (g_m), a critical parameter for a transistor, is obtained by taking the derivative of Eq. (2) with respect to V_{GS}. It evaluates the better switching capacity and operational efficiency of the device, which can be expressed as Equation (4)

$$g_m = \frac{\partial I_{DS}}{\partial V_{GS}} \qquad (4)$$

The field effect mobility Equation (5) can be determined by calculating the transconductance (g_m) provided by

$$\mu_{eff} = \frac{Lg_m}{wC_i} \times \frac{1}{V_{DS}} \qquad (5)$$

Thus, the carrier mobility in the saturation region can be computed from Equation (3) as

$$\mu_{sat} = \frac{2L}{WC_i}\left(\frac{d\sqrt{I_{DS}}}{dV_{GS}}\right)^2 \qquad (6)$$

Simulated Approach

The schematic of the proposed 2D dimensional bottom gate top contact DNTT-based OTFT device with the Al_2O_3/PVP bilayer dielectric structure with dimensions of 1 µm channel length (L) and 200 µm width (W) utilized in this simulation is illustrated in Fig. 2(a). The aluminium with a thickness of 30 nm can act as a gate as well as a substrate for the device. Next, deposit high-K Al_2O_3 over the gate electrode, and organic PVP can be covered over the Al_2O_3 to provide a suitable surface for uniform deposition of the organic semiconductor. The total thickness of the hybrid dielectric layer is 12nm. Then an organic semiconductor, DNTT, is deposited over the top of the PVP layer with a thickness of 25 nm, thus providing an organic-organic interface. Finally, deposit gold material of 30 nm as a source and drain contact on the layer of organic semiconductor. The

dimensions and materials are adopted from the reference [27]. The simulation utilized a comprehensive list of geometric and device parameters, as detailed in Table 1. The performance of the DNTT-based OTFT is investigated by varying the single and hybrid dielectric layers of Al_2O_3 and PVP with the help of Silvaco atlas device simulator. The overall thickness of dielectric layer maintains constant throughout the simulations but varied the ratio of thickness composition of Al_2O_3 and PVP in the hybrid dielectric structures, that namely as P5A95, P20A80, and P40A60 as shown in Fig. 2. The Silvaco, a numerical simulator, analyzes the direct current (DC) and transient behavior of a device. This tool predicts and optimizes the performance of electronic devices and circuits prior to fabrication. In the simulation process, the structure is discretized into a mesh of triangular nodes. The selection of a fine mesh achieves an optimal balance between precision and computational efficiency, as shown in Fig. 2(a). The 2D ATLAS Silvaco tool performs numerical analysis by solving self-consistent Poisson and drift-diffusion equations at each mesh node.

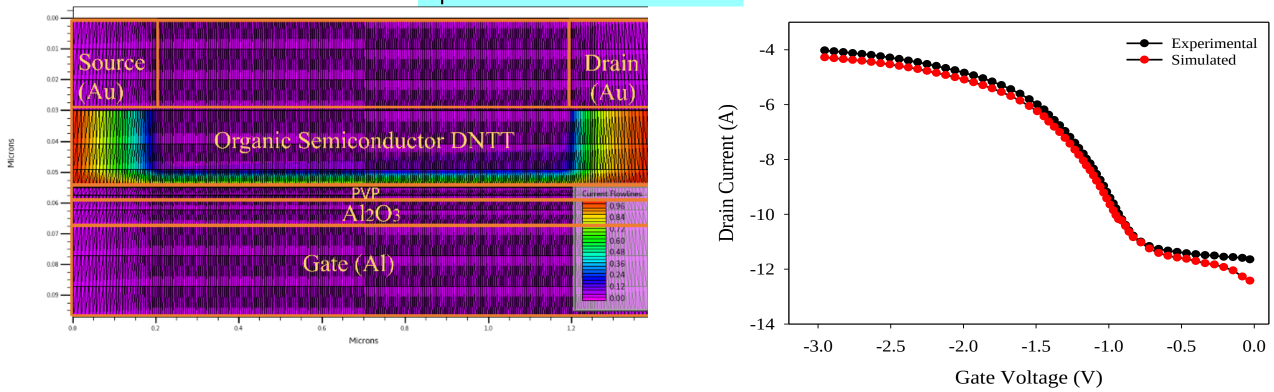

(a) (b)

Fig 2. (a) Simulated structure of top contact bilayer gate dielectric OTFT representing current flow lines at conducting channel (b) Comparison of experimental and simulated transfer characteristics of OTFT at 300K [27]

To model the DNTT layer, the simulation incorporates the Langevin recombination concept, Shockley-Read-Hall (SRH) processes, trap states, as well as Poole-Frenkel and hopping mobility. The PF mobility model establishes the relationship between mobility capabilities and the interaction with the electrical field. This model is mathematically represented as [29].

$$\mu(E) = \mu(0)exp\left(\frac{\beta\sqrt{E}}{KT}\right) \qquad (7)$$

Where the zero field mobility,

$$\mu(0) = \mu_i exp\left(-\frac{\Delta}{KT}\right) \qquad (8)$$

where $\mu(E)$ is the field dependent mobility, β is the Poole–Frenkel factor, E is the electric field, Δ is the zero field thermal activation energy, and μ_i is the intrinsic mobility. Table 1 enumerates the material input parameters for hybrid dielectric OTFT used in the TCAD simulation.

In this study, we performed device simulations assuming a trap-free interface at the dielectric-semiconductor boundary to evaluate key device parameters such as the subthreshold swing (SS), on/off current ratio, and transconductance (gm). The interface trap density can be expressed as Eq. (9)

$$N_{it} = \left(\frac{SS}{ln10}\frac{q}{KT} - 1\right)\frac{C_{ox}}{q} \qquad (9)$$

Where N_{it} is directly proportional to the gate capacitance and SS.

It is important to note that the actual interface trap density can vary depending on the fabrication method, material quality, and processing conditions. These factors significantly influence the interfacial properties, such as charge trapping and mobility degradation, and consequently impact the overall device performance.

Table 1. Summary of material parameters used in simulation

Material	Parameter	Value
Al_2O_3	Dielectric Constant	9.3 [30]
PVP	Dielectric Constant	3.8
Gold	Work Function	5.0 eV
Aluminium	Work Funtion	4.28 eV
Organic Semiconductor	Bandgap (eg)	3.38 [31]
	Permittivity (ε)	3
	Electron Affinity(χ_e)	1.81
	Effective DOS of LUMO (N_c)	$1x10^{20}$ cm^{-3}
	Effective DOS of HOMO(N_v)	$1x10^{20}$ cm^{-3}
	Doping Concentration	$1x10^{15}$ cm^{-3}

In order to achieve the calibration between the simulated and fabricated OTFTs, the density of state parameters was adjusted to W_{TA} = 0.013, W_{TD} = 0.12, N_{GA} = $1.2x10^{14}$, N_{GD} = $2x10^{16}$, E_{GA} = 1.2, E_{GD} = 2.9, and W_{GA} = 0.05. The graph shows that there is a slight variation in the calibration results, particularly at the zero voltage of V_{GS}. This happened as a result of the reduction in trap densities caused by the hybrid dielectric layer. However, it is important to note that the device slope and the ON current at V_{DS} = -1.5V substantially resemble those of the fabricated device reported by Kraft, Ulrike, et al. [27], as shown in Fig. 2(b). Therefore, it may be concluded that the simulation setup is reliable and can be further employed.

Results and Discussion

Selecting the appropriate dielectric material significantly influences the performance of thin-film transistor (TFT) devices. To investigate the interfacial performance of OTFTs, we first introduced an Al_2O_3 dielectric layer with a fixed interface trap density of 5×10^{-11} cm^{-2}. This layer was then replaced with a hybrid Al_2O_3/PVP dielectric, which has a lower interface trap density, in order to improve the dielectric/semiconductor interface. The hybrid dielectric reduces trap density at the interface, preventing trap formation and minimizing leakage current through the dielectric [32]. This study implements a bilayer dielectric configuration to enhance both the performance and reliability of the device.

OTFT with Single Dielectric layer of Al_2O_3 and PVP

Figure 3. shows the transfer and output characteristics of a DNTT-based OTFT with single dielectric layers of Al_2O_3 and PVP. We evaluated the electrical parameters from both the linear and saturation regions of the transfer characteristics, as extracted from equations (1–6), and summarized them in Table 3. The threshold voltage was measured from the transfer characteristics by plotting the square root of the drain current versus gate voltage, as shown in Figures 3a and 3b. The OTFT with a single Al_2O_3 dielectric layer exhibits a lower threshold voltage compared to the PVP-based OTFT, which can be attributed to the higher dielectric capacitance of Al_2O_3. This higher capacitance allows more charge carriers to accumulate at the interface between the semiconductor and dielectric layers, even at low gate voltages.

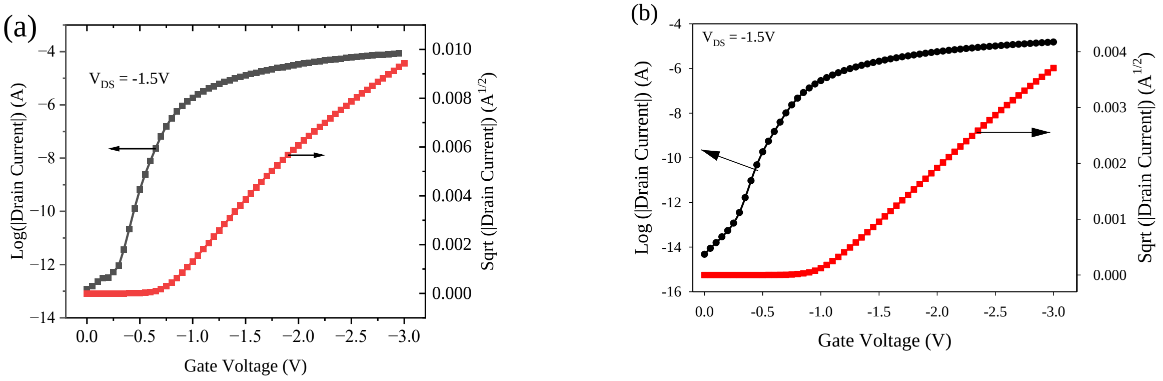

Fig 3. Transfer characteristics of OTFT with single dielectric of (a) Al_2O_3 and (b) PVP

However, despite this advantage of low threshold voltage, the Al_2O_3-based OTFT also demonstrates certain limitations, such as lower mobility (3.86 $cm^2/V\cdot s$) and a higher leakage current density of 10^{-6} A/cm^2. These drawbacks are likely due to the high surface energy at the interface and the inherent characteristics of Al_2O_3, such as its high density of random dipoles at the semiconductor-dielectric interface. According to Veres et al., these dipoles can disorganize the energy landscape, leading to reduced field-effect mobility in OTFTs [33]. On the other hand, employing an organic dielectric such as PVP, which has a smooth surface and low surface energy, can overcome some of these disadvantages. The low surface energy of PVP reduces interface traps and charge carrier scattering at the semiconductor interface, facilitating easier electron movement [32]. However, despite these advantages, PVP has a low dielectric constant, which necessitates high operating voltages. Table 3 summarizes the electrical characteristics of the OTFTs with single dielectric layers of Al_2O_3 and PVP.

To address the limitations of single dielectrics, a hybrid bilayer dielectric combining Al_2O_3 and PVP provides a promising solution for enhancing OTFT performance. By incorporating a thin layer of low-k organic dielectric (PVP) over a high-k inorganic dielectric (Al_2O_3) the hybrid structure effectively reduces leakage current density, improves the ION/IOFF ratio, and enhances switching performance, thereby reducing power consumption. As shown in Fig. 4, the J-V characteristics on a logarithmic scale highlight the advantages of the hybrid dielectric. The Al_2O_3 /PVP devices exhibit a significantly reduced leakage current density of 10^{-8} A/cm^2 at -3V, which is two orders of magnitude lower than Al_2O_3 and one order of magnitude lower than PVP. At VDS = -0.05V, thus the bilayer dielectric demonstrates minimal leakage current, enhancing device reliability. These results underscore the hybrid dielectric's potential to overcome the trade-offs associated with single dielectrics, offering a balanced approach to improved device performance and stability.

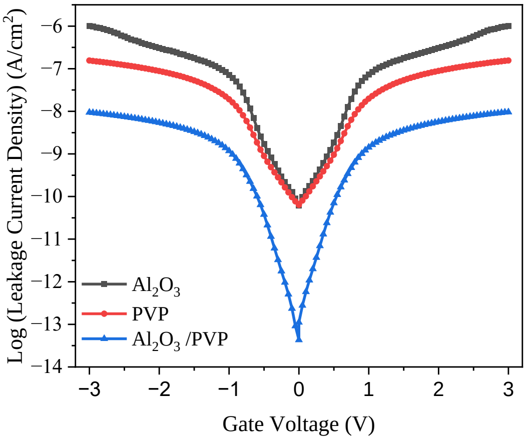

Fig 4. Shows the leakage current density-voltage (J-V) for single and hybrid dielectric of Al_2O_3/PVP

Effect of hybrid Dielectric layer of Al_2O_3/ PVP

Figure. 5 shows the C-V characteristics of MIS capacitors with an Al_2O_3/PVP hybrid dielectric, using various thickness compositions. The following Equation (10) can be used to calculate a bilayer dielectric's gate capacitance.

$$\frac{1}{C_i} = \frac{1}{C_{High-k}} + \frac{1}{C_{Low-k}} \qquad (10)$$

C_i denotes the overall capacitance of the bilayer, whereas C_{High-k} and C_{Low-k} represent the capacitance of the high-k and low-k dielectric layers, respectively. The capacitance densities for the compositions P5A95, P20A80, and P40A60 are 12.78 nF/cm² (K=8.7), 10.64 nF/cm², and 9.56 nF/cm² (K=6.4), respectively.

As the PVP composition in the hybrid dielectric increases, we observed a gradual decrease in the capacitance due to a reduction in the overall dielectric constant. The lower capacitance results in higher gate voltages being required to accumulate charge carriers at the dielectric interface, which shifts the depletion region toward the positive.

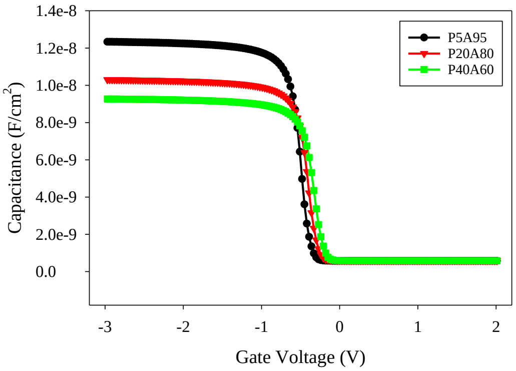

Fig 5. The C-V characteristics of the various thickness composition of Al_2O_3 /PVP at 1MHz.

This shift can lead to an increase in the threshold voltage. The dielectric constants of single and hybrid dielectric layers with various compositions of Al_2O_3 and PVP are evaluated based on capacitance density as per Equation (11) and listed in Table 2.

$$\varepsilon_r = \frac{C_{ox}.\,t_{die}}{\varepsilon_0} \qquad (11)$$

Where t_{die} represents the total thickness of hybrid dielectric layer and C_{ox} represents gate dielectric capacitance.

Table 2. Dielectric Properties of the Single and Bilayer Dielectrics

Dielectric Materials	Dielectric Constant
Single Al_2O_3	9.3
Single PVP	3.8
P5A95	8.66
P20A80	7.2
P40A60	6.4

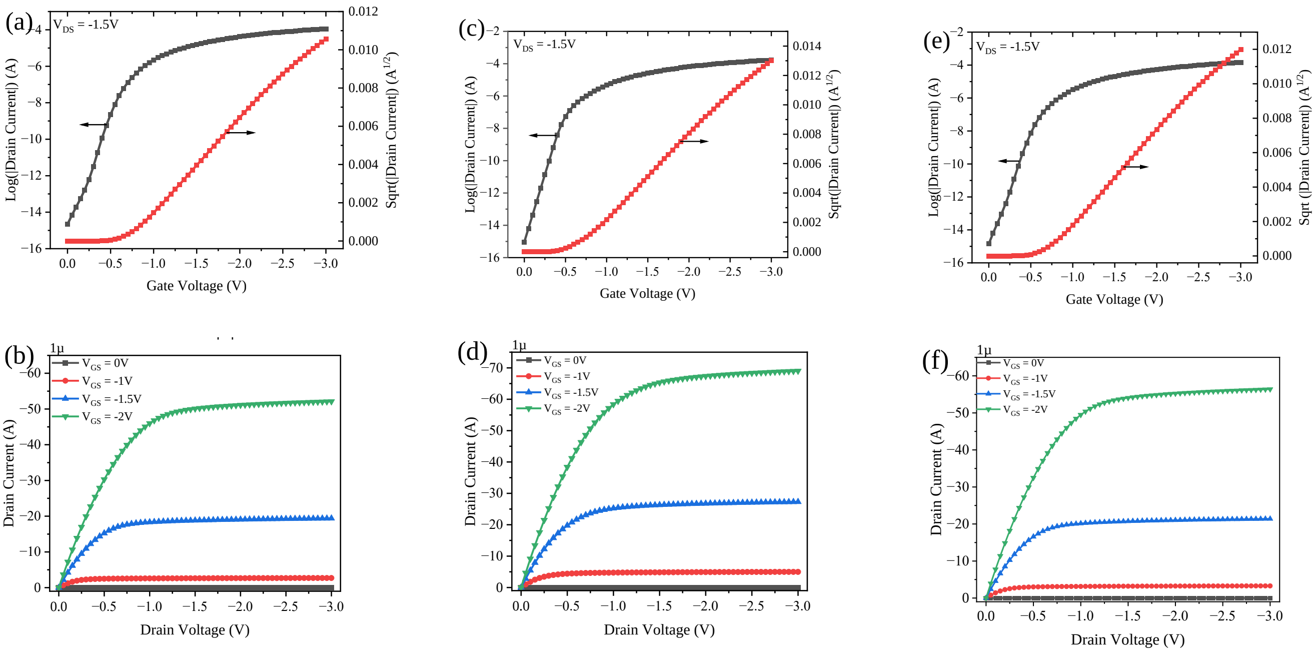

Fig 6. Transfer and output characteristics of OTFT with various composition of (a) P5A95. (b) P20A80. (c) P40A60.

The transfer and output characteristics of DNTT-based OTFTs with hybrid dielectrics (Al_2O_3/PVP) were analyzed by varying the composition ratios of Al_2O_3 to PVP, namely P5A95, P20A80, and P40A60, while maintaining a constant dielectric thickness of 12 nm, as shown in Figure 6. From the output characteristics, it can be observed that all devices exhibit clear linear and saturation regions at a gate voltage of -3 V. The transfer characteristics indicate that increasing the PVP composition in the hybrid dielectric—such as in P5A95 and P20A80 devices—leads to a gradual increase in drain current. This improvement is attributed to enhanced interface quality, despite the reduced gate capacitance. Additionally, the OFF current decreases with higher PVP content, providing better isolation between the electrodes and the conducting channel, which increases the I_{ON}/I_{OFF} ratio (up to 1.83 × 10^{10}), making the devices suitable for high-speed applications.

The threshold voltage (V_{TH}) was extracted from the plot of versus V_{GS}, and the subthreshold swing (SS) was obtained from the transfer characteristics of the OTFT at V_{DS} = −1.5Vas shown in Figure 5. The performance parameters, including SS and V_{TH}, are critical for improving fast switching and lowering the operating voltage of the device. As the PVP composition in the hybrid dielectric increases, the values of SS and V_{TH} were found to be 85 mV/dec and 0.65 V for P5A95, 83 mV/dec and 0.6 V for P20A80, and 84 mV/dec and 0.75 V for P40A60. It was observed that further increasing the PVP thickness results in a higher threshold voltage and lower drain current due to reduced capacitance. A thinner PVP composition in the hybrid dielectric minimizes carrier scattering and reduces interface trap density, enhancing mobility (up to 8.43 cm^2/Vs).
However, when the PVP composition exceeds 35%, carriers are trapped at the interface, reducing capacitance and mobility (8.17 cm^2/V·s). From our results, the optimal PVP composition in the hybrid dielectric OTFT ranges from 20% to 35% (2.4 nm to 4.2 nm) to prevent carrier trapping at the interface and avoid significant reductions in capacitance and increases in operating voltage. The transfer characteristics of the P20A80 hybrid dielectric OTFT show a notable increase in drain current (1.69 × 10^{-4} A), surpassing the performance of OTFTs with single Al_2O_3 or PVP dielectrics by approximately one order of magnitude.

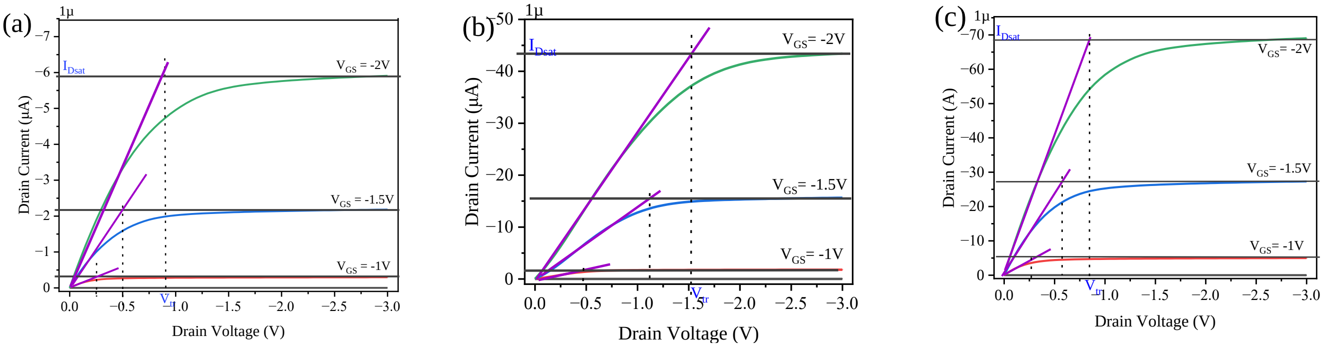

Fig 7. The contact resistance has been extracted from the output characteristics of OTFT (a)PVP (b) Al_2O_3 (c) Al_2O_3/PVP dielectrics

Alghamdi and Noweir Ahmad [34] developed the transition voltage method (TVM) to extract the contact resistance (R_c) from the output characteristics of an OTFT when it is biased in the saturation region. The contact resistance was measured at different values of V_{GS} and expressed in Equation (12).

$$R_C = 2\frac{\sqrt{V_{GS}-V_{TH}}\left[\sqrt{2V_{tr}}-\sqrt{V_{GS}-V_{TH}}\right]}{I_{DSAT}} \qquad (12)$$

I_{DSAT} represents the saturation current, while V_{tr} represents the voltage at which the OTFT transitions from the linear to the saturation region. Therefore, the TVM method is applicable to all devices that exhibit a transition from linear to saturation. Figure 7. shows how the transition voltage method (TVM) is used to extract the contact resistance from the output characteristics of OTFTs with single and hybrid dielectrics. It was observed that hybrid dielectrics exhibit a lower transition voltage compared to single dielectrics, meaning that the transition from the linear to saturation region occurs at lower drain voltages. The contact resistance is significantly reduced to 4.8 KΩ in OTFTs with an Al_2O_3/PVP hybrid dielectric. This reduction is attributed to fewer traps and defects at the interface, which decreases the Schottky barrier height and allows charge carriers to be injected more easily into the channel from the electrodes.

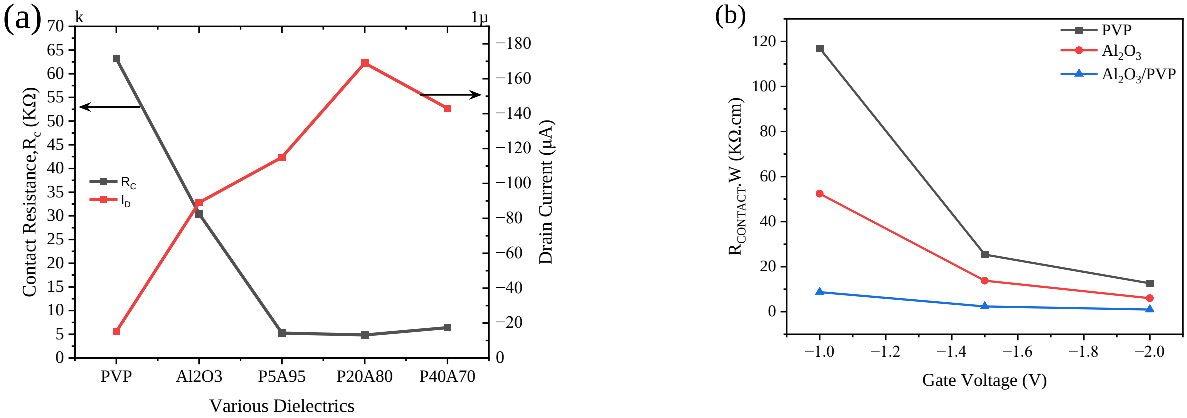

Fig 8. (a) Impact of Contact resistance on drain current (b) Rc.W at different V_{GS} for single and hybrid dielectrics of OTFT.

Figure 8(a) illustrates the impact of contact resistance on the drain current in OTFTs with both single and hybrid dielectrics, demonstrating how reduced contact resistance enhances current flow. Figure 8(b) compares the extracted

contact resistance (R_c.W) of top-contact OTFTs with single and hybrid dielectrics of Al_2O_3 and PVP. The contact resistance decreases gradually with increasing gate voltage, which is consistent with previous studies [35-36]. Notably, the hybrid dielectric of Al_2O_3/PVP reduces R_c more effectively than single dielectric layers, with Rc extracted at V_{GS} = -2 V decreasing from 63.5 KΩ to 4.8 KΩ. This reduction in contact resistance improves the overall functionality of the device. In this work, we observe that the decrease in contact resistance is primarily due to the reduction in carrier dispersion, improved interface quality, lower trap density, and enhanced charge carrier injection from the electrodes. Optimizing the PVP composition and thickness in the Al_2O_3/PVP hybrid dielectric of OTFTs is a promising approach to improving device performance and reducing contact resistance. Figure 9(a) shows the I_{ON} and I_{OFF} values for OTFTs with single and hybrid dielectrics. The I_{ON} increases from -1.515×10^{-5} A to -1.69×10^{-4} A with hybrid dielectrics, compared to single dielectric layers, due to reduced contact resistance and improved interface quality. Additionally, the I_{OFF} is reduced in hybrid dielectric OTFTs, leading to an I_{ON}/I_{OFF} ratio of 1.83×10^{10}, which is suitable for high-speed applications. The transconductance (g_m) is a key parameter for evaluating device linearity, power efficiency, and amplification capabilities in OTFTs. As shown in Figure 9(b), in single dielectrics (Al_2O_3 and PVP), transconductance increases with gate bias until a peak is reached, after which it degrades due to increased charge scattering at higher voltages. This effect is minimized by employing a hybrid dielectric layer (Al_2O_3/PVP), which reduces interface trap states at the dielectric-semiconductor interface, resulting in better device stability and performance.

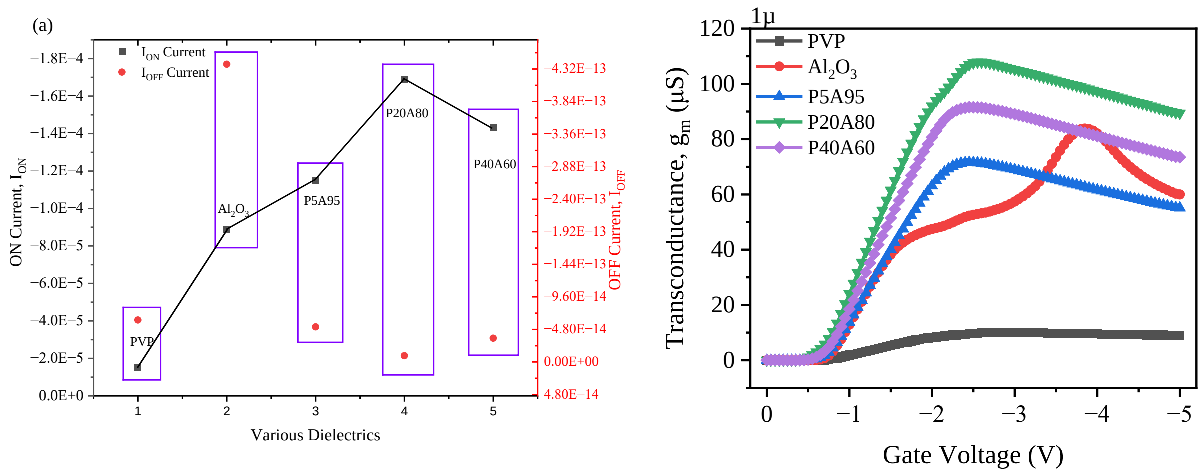

Fig 9. (a) I_{ON} versus I_{OFF} (b) transconductance of single and various hybrid dielectric compositions

Table 3. Electrical Performance Parameters of DNTT- based OTFT with single and hybrid dielectrics

**Device Characteristics**	**Single Layer**		**Hybrid Layer composition**		
	Al_2O_3	PVP	P5A95	P20A80	P40A60
V_{TH}(V)	-0.8	-0.5	-0.65	-0.6	-0.75
I_{ON} (A)	-1.51×10^{-5}	-8.90×10^{-5}	-1.15×10^{-4}	-1.69×10^{-4}	-1.43×10^{-4}
I_{OFF} (A)	-6.19×10^{-14}	-4.39×10^{-13}	-5.17×10^{-14}	-9.24×10^{-15}	-3.50×10^{-14}
I_{ON}/I_{OFF} ratio	2.44×10^{8}	2.03×10^{8}	2.22×10^{9}	1.83×10^{10}	4.08×10^{9}
SS (mV/dec)	100	96	85	83	84
G_m(S)	1.01×10^{-5}	5.73×10^{-5}	6.89×10^{-5}	1.05×10^{-4}	8.90×10^{-5}
C_i[nF/cm^2]	5.6	13.7	12.78	10.64	9.56
Mobility sat(cm^2/Vs)	5.16	3.86	5.28	8.43	8.17
Contact Resistance (KΩ)	63.10	30.04	5.07	4.84	6.13
Interface trap density (N_{it}) cm^{-2}	2.39×10^{10}	5.24×10^{10}	3.42×10^{10}	2.62×10^{10}	2.85×10^{10}

Effect of Interfacial Properties of Single and hybrid Dielectrics of Al_2O_3/PVP

The efficiency of OTFTs is significantly influenced by the selection of the dielectric layer. To enhance the performance and reliability of the device, we have employed a bilayer dielectric structure consisting of Al_2O_3 and PVP. This arrangement incorporates a thin layer of PVP to provide a low interface trap density at the channel interface, while a larger high-k Al_2O_3 layer (K = 9.3) enhances gate capacitance and facilitates low-voltage operation. The integration of these layers optimizes the advantages of a high dielectric constant and diminished interfacial traps, as demonstrated in Fig. 10. To further understand the relationship between interfacial properties and device performance, we evaluated leakage current density, C-V characteristics, and hysteresis loss in OTFTs with interface trap densities of 5.24×10^{10} cm^{-2}, 2.39×10^{10} cm^{-2}, and 2.62×10^{10} cm^{-2} for single dielectrics ($Al_2O_3$and PVP) and the hybrid dielectric (Al_2O_3/PVP), respectively. The C-V characteristics in Figure. 11(a) demonstrate that the single Al_2O_3 dielectric exhibits a negative flat band voltage (V_{FB}) of -0.5V, primarily due to the presence of high density of interface traps at the dielectric-semiconductor interface. In contrast, the organic dielectric PVP provides a smoother surface interface, resulting in a more positive flat band voltage of 0.4V. PVP's lower capacitance is attributed to its inherently low dielectric constant. By employing hybrid dielectrics (Al_2O_3/PVP), the properties of both organic and

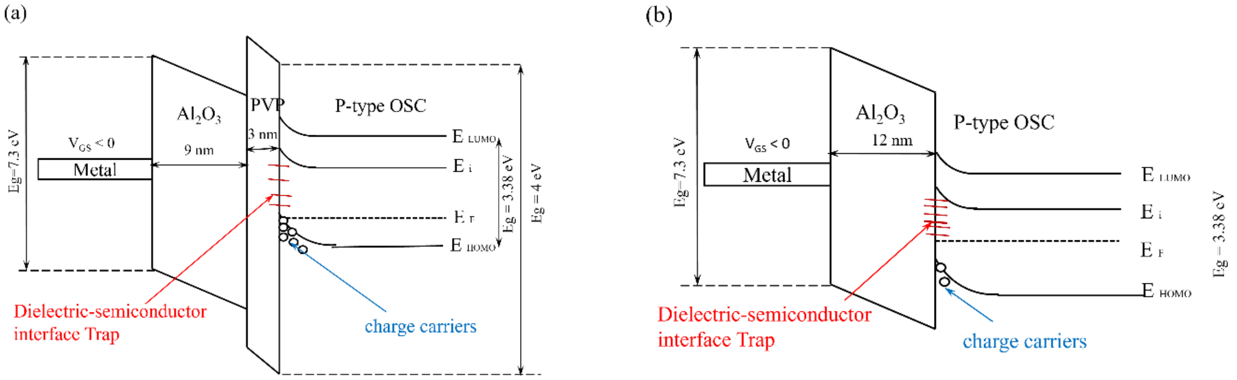

Fig 10. Schematic band energy diagram of the dielectric-semiconductor interface in a DNTT based OTFT with (a) Al_2O_3/PVP gate hybrid dielectric, (b) single Al_2O_3 dielectric

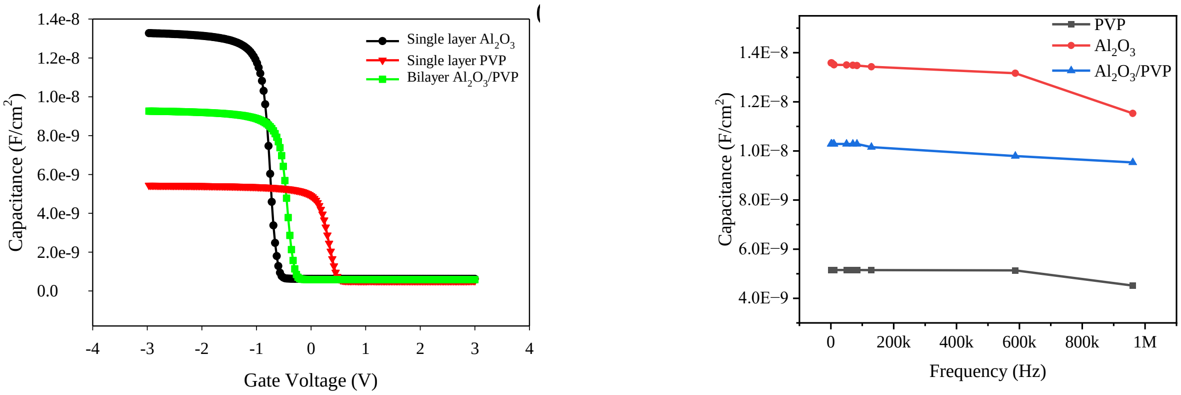

Fig 11. (a) capacitance-voltage (CV), and (b) capacitance-frequency (C-F) characteristics for single and hybrid dielectric of Al_2O_3/PVP

inorganic dielectrics are combined, leading to intermediate values of flat band voltage and capacitance. Specifically, the flat-band voltage shifts to -0.1 V, indicating a reduced density of interface traps and an improved interface quality. Additionally, the capacitance is improved to 10 nF, enhancing overall device performance. Thus, the hybrid dielectric OTFT can operate at lower voltages, making it suitable for applications in wearable electronics and flexible electronics.

Furthermore, as seen in Figure 11(b), the dielectric stability can be investigated by examining the C-F characteristics of various dielectrics of both single Al_2O_3 and PVP, as well as hybrid dielectric layers of Al_2O_3 and PVP. As observed, the hybrid dielectric OTFT has demonstrated that the capacitance remains constant over the 1 MHz frequency in comparison to single dielectric layers. This stable capacitance over a wide range of frequencies can ensure the reliability and robustness of the hybrid dielectric layer, contributing to the long-term performance of the device. Figures 12(a) and 12(b) illustrate the electric field distribution across the single Al_2O_3 layer and the hybrid Al_2O_3/PVP layer. The results indicate that the hybrid dielectric improves interface quality, leading to the development of a higher electric field at the interface. This stronger electric field facilitates better carrier injection and reduces carrier scattering, resulting in a higher current density across the channel compared to the single Al_2O_3 dielectric. Figures 12(c) and 12(d) show the total current density across the conducting channels of the single Al_2O_3 and hybrid Al_2O_3/PVP dielectrics. These visual representations provide a comprehensive understanding of the performance parameters of the devices.

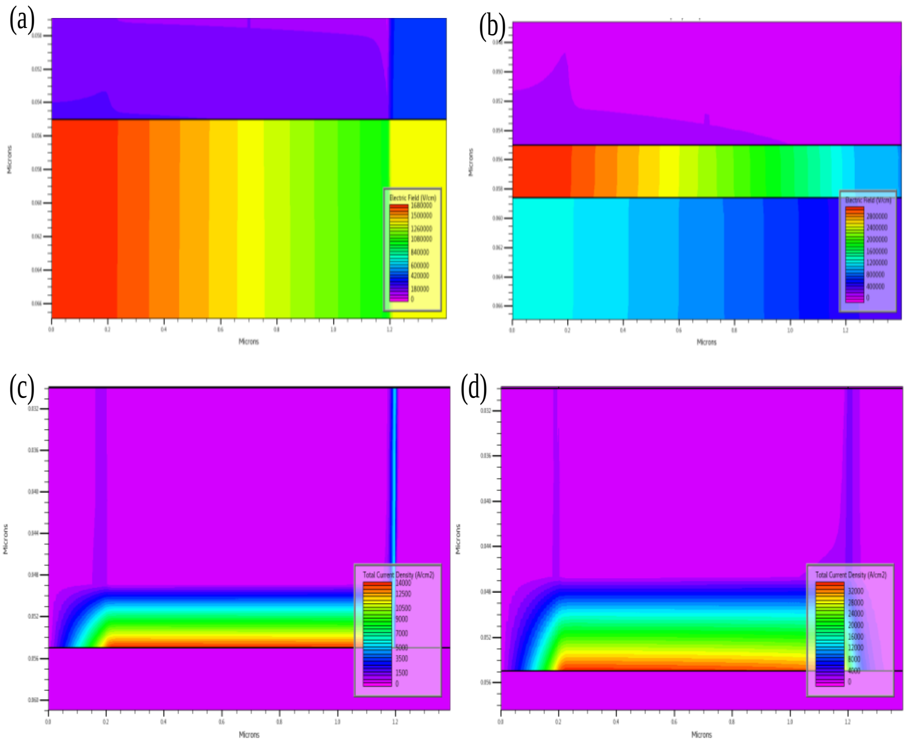

Fig 12. The contour plots of electric fields of (a) single Al_2O_3 and (b) hybrid Al_2O_3/ PVP and total current density of (c) single Al_2O_3 and (d) hybrid Al_2O_3 at V_{DS} = -1.5V

To further investigate hysteresis loss in OTFTs with single and hybrid dielectrics, we measured the C-V curves in two opposite gate voltage sweep directions, as shown in Figure 13. The gate voltage sweeps ranged from −3 V to +3 V and back to −3 V. In the forward sweep, the C-V curves for both dielectrics shift towards the negative voltage direction, primarily due to hole trapping. During the

backward sweep, the C-V curves for the single-dielectric Al_2O_3 exhibit a shift towards the positive direction, resulting from strong charge carrier trapping at

Table 4. Comparison of electrical performances organic TFTs based on various channel lengths and hybrid dielectrics

Refrences	Hybrid dielectric	Organic Semiconductor	Channel Length (µm)	I_{ON}/I_{OFF} Ratio	SS(V/dec)	Threshold Voltage(V)	Operating Voltage (V)
[37]	$PMMA/Ta_2O_5$	Pentacene	100	$4.3x10^4$	1.04	-3.5	0 to -20V
[38]	PVP/Al_2O_3	Pentacene	90	$5x10^5$	0.5	-5	0 to -10V
[20]	$PMMA/ZrO_2$	Copper ph-thalocyanine (CuPc)	60	$1.2x10^3$	2.1	-0.8	0 to -40V
[39]	KPSPI-6F/PVP	Pentacene	50	$1.7x10^5$	0.2	-3.4	0 to -5 V
This work	PVP/Al_2O_3	DNTT	1	$1.83x10^{10}$	0.083	-0.6	0 to -3V

the interface. This phenomenon leads to a significant hysteresis loop of 0.64 V for the Al_2O_3 dielectric. In contrast, the hybrid dielectric shows minimal shift in the C-V curves, as indicated in Figure 13(b). This is attributed to the reduced charge trapping at the interface, due to the improved organic-organic interface characteristics. The hysteresis loss for the Al_2O_3/PVP hybrid dielectric is as small as 0.02 V, which is negligible. This reduction in hysteresis underscores the effectiveness of the hybrid dielectric in facilitating charge carrier movement from the electrodes to the active semiconductor, largely due to the lower contact resistance.

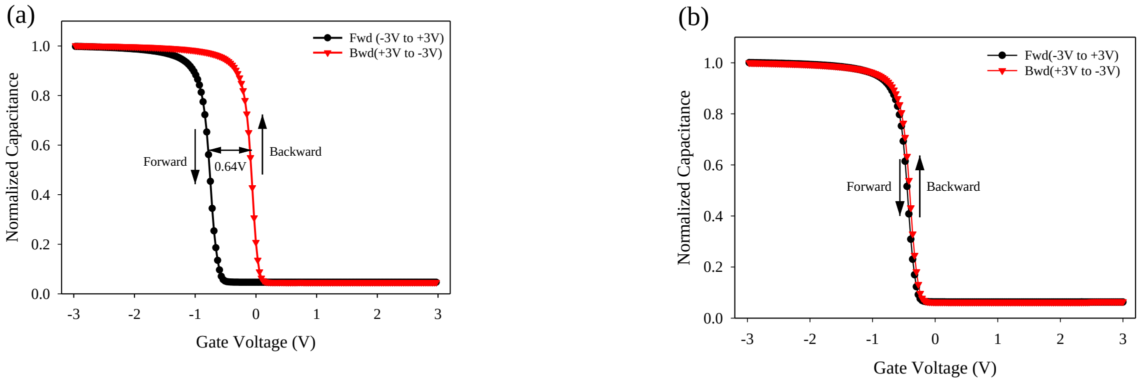

Fig 13. C-V hysteresis curves of (a)single Al_2O_3 (b) Al_2O_3/PVP as hybrid dielectrics for OTFT at 1MHz

The short-channel OTFT, utilizing a hybrid dielectric of Al_2O_3 and polyvinyl phenol (PVP), exhibits improved performance over other hybrid dielectrics by effectively controlling contact resistance and optimizing dielectric dimensions. A summary of these comparisons is provided in Table 4. The short channel (1 µm) OTFTs offers significant advantages for next-generation flexible and high-performance electronics by leveraging the high dielectric constant of Al_2O_3 and the organic interface

properties of PVP. This combination results in superior interface quality, leading to a steeper subthreshold slope of 83 mV/dec, a higher I_{ON}/I_{OFF} ratio, and a lower threshold voltage, making it suitable for low-power applications compared to other dielectrics. These improvements make the OTFT particularly well-suited for use in biosensors, where high sensitivity and stability are crucial for detecting biological molecules, as well as in high-speed electronics, where it can operate effectively at higher frequencies. This innovation not only enhances current applications but also opens possibilities for future developments in wearable electronics and flexible displays.

Conclusion

In this paper, we simulated and analyzed a top-contact, bottom-gate OTFT using both single and hybrid dielectric materials, specifically Al_2O_3 and PVP. We examined how the interfacial properties of single and hybrid dielectric layers affect the OTFT's performance. Optimizing the PVP thickness in the hybrid layer was found to enhance device performance by reducing contact resistance (R_c) in short-channel OTFTs. The optimal PVP thickness was identified as 2.4 nm to 4.2 nm; beyond this range, further increases in PVP thickness led to reduced capacitance and higher operating voltage. Additionally, R_c was shown to significantly impact drain current and charge carrier mobility. The bilayer dielectric also mitigated the hysteresis loss commonly observed with single Al_2O_3 dielectrics. Our findings offer a straightforward approach to minimizing short-channel effects, making these devices well-suited for low-voltage, flexible electronics applications such as biosensors and disposable electronics like smart packaging.

References

1. Liu K, Ouyang B, Guo X, Guo Y, Liu Y. Advances in flexible organic field-effect transistors and their applications for flexible electronics. npj Flexible Electronics. 2022 Jan 20;6(1):1.
2. Ryu GS, Kim JS, Jeong SH, Song CK. A printed OTFT-backplane for AMOLED display. Organic Electronics. 2013 Apr 1;14(4):1218-24.
3. Baude PF, Ender DA, Haase MA, Kelley TW, Muyres DV, Theiss SD. Pentacene-based radio-frequency identification circuitry. Applied Physics Letters. 2003 Jun 2;82(22):3964-6.
4. Baeg KJ, Khim D, Kim J, Yang BD, Kang M, Jung SW, You IK, Kim DY, Noh YY. High-performance top-gated organic field-effect transistor memory using electrets for monolithic printed flexible NAND flash memory. Advanced Functional Materials. 2012 Jul 24;22(14):2915-26.
5. Someya T, Sekitani T, Iba S, Kato Y, Kawaguchi H, Sakurai T. A large-area, flexible pressure sensor matrix with organic field-effect transistors for artificial skin applications. Proceedings of the National Academy of Sciences. 2004 Jul 6;101(27):9966-70.
6. Rogers JA, Bao Z, Baldwin K, Dodabalapur A, Crone B, Raju VR, Kuck V, Katz H, Amundson K, Ewing J, Drzaic P. like electronic displays: Large-area rubber-stamped plastic sheets of electronics and microencapsulated electrophoretic inks. Proceedings of the National Academy of Sciences. 2001 Apr 24;98(9):4835-40.
7. Fan, Ching-Lin, et al. "Effects of the F4TCNQ-doped pentacene interlayers on performance improvement of top-contact pentacene-based organic thin-film transistors." *Materials* 9.1 (2016): 46.
8. Lee CT, Chen HC. Performance improvement mechanisms of organic thin-film transistors using MoOx-doped pentacene as channel layer. Organic Electronics. 2011 Nov 1;12(11):1852-7.
9. Roh J, Lee C, Kwak J, Jung BJ, Kim H. Vapor-phase-processed fluorinated self-assembled monolayer for organic thin-film transistors. Journal of the Korean Physical Society. 2015 Sep;67:941-5.
10. Lee J, Park JS, Lee BL, Park JI, Chung JW, Lee S. Ionic self-assembled monolayer for low contact resistance in inkjet-printed coplanar structure organic thin-film transistors. Organic Electronics. 2014 Sep 1;15(9):2021-6.
11. Wondmagegn WT, Satyala NT, Pieper RJ, Quevedo-Lopez MA, Gowrisanker S, Alshareef HN, Stiegler HJ, Gnade BE. Impact of semiconductor/metal interfaces on contact resistance and operating speed of organic thin film transistors. Journal of computational electronics. 2011 Jun;10:144-53.

12. Waldrip M, Jurchescu OD, Gundlach DJ, Bittle EG. Contact resistance in organic field-effect transistors: conquering the barrier. Advanced functional materials. 2020 May;30(20):1904576.
13. Hyung GW, Park J, Koo JR, Li ZH, Kwon SJ, Cho ES, Kim YK. Improved performance of pentacene thin-film transistors with Al2O3 gate dielectric: annealing effect on the surface properties. Japanese Journal of Applied Physics. 2012 Jan 20;51(2R):025702.
14. Yoon WJ, Berger PR. Atomic layer deposited HfO2 gate dielectrics for low-voltage operating, high-performance poly-(3-hexythiophene) organic thin-film transistors. Organic Electronics. 2010 Nov 1;11(11):1719-22.
15. Bartic C, Jansen H, Campitelli A, Borghs S. Ta2O5 as gate dielectric material for low-voltage organic thin-film transistors. Organic electronics. 2002 Jun 1;3(2):65-72.
16. He W, Xu W, Peng Q, Liu C, Zhou G, Wu S, Zeng M, Zhang Z, Gao J, Gao X, Lu X. Surface modification on solution processable ZrO2 high-k dielectrics for low voltage operations of organic thin film transistors. The Journal of Physical Chemistry C. 2016 May 12;120(18):9949-57.
17. Lu Y, Lee WH, Lee HS, Jang Y, Cho K. Low-voltage organic transistors with titanium oxide/polystyrene bilayer dielectrics. Applied Physics Letters. 2009 Mar 16;94(11).
18. J.J.SenkevichandS et al. ,Poly(tetra-fluoro-p-xylylene).Appl.Phys.Lett1998,72,258ayer dielectrics." *Applied Physics Letters* 94.11 (2009).
19. Hulea IN, Fratini S, Xie H, Mulder CL, Iossad NN, Rastelli G, Ciuchi S, Morpurgo AF. Tunable Fröhlich polarons in organic single-crystal transistors. Nature materials. 2006 Dec 1;5(12):982-6.
20. Shang L, Liu M, Tu D, Liu G, Liu X, Ji Z. Low-Voltage Organic Field-Effect Transistor With PMMA/$\hbox {ZrO} _ {2} $ Bilayer Dielectric. IEEE transactions on electron devices. 2009 Jan 27;56(3):370-6.
21. Khound S, Sarmah JK, Sarma R. Hybrid La2O3-cPVP dielectric for organic thin film transistor applications. ECS Journal of Solid State Science and Technology. 2022 Jan 21;11(1):013007.
22. Paterson AF, Mottram AD, Faber H, Niazi MR, Fei Z, Heeney M, Anthopoulos TD. Impact of the gate dielectric on contact resistance in high-mobility organic transistors. Advanced Electronic Materials. 2019 May;5(5):1800723.
23. Liu C, Xu Y, Li Y, Scheideler W, Minari T. Critical impact of gate dielectric interfaces on the contact resistance of high-performance organic field-effect transistors. The Journal of Physical Chemistry C. 2013 Jun 13;117(23):12337-45.
24. Jo SW, Cho S, Kim CH. Key factors affecting contact resistance in coplanar organic thin-film transistors. Journal of Physics D: Applied Physics. 2022 Jul 25;55(40):405101.
25. Wondmagegn WT, Satyala NT, Pieper RJ, Quevedo-Lopez MA, Gowrisanker S, Alshareef HN, Stiegler HJ, Gnade BE. Impact of semiconductor/metal interfaces on contact resistance and operating speed of organic thin film transistors. Journal of computational electronics. 2011 Jun;10:144-53.
26. Nirosha R, Agarwal R. Comprehensive Temperature-Dependent DC Characterization of Organic Thin Film Transistor for Sensing Applications. IEEE Sensors Journal. 2022 Jul 27;22(17):16794-803.
27. Kraft U, Takimiya K, Kang MJ, Rödel R, Letzkus F, Burghartz JN, Weber E, Klauk H. Detailed analysis and contact properties of low-voltage organic thin-film transistors based on dinaphtho [2, 3-b: 2′, 3′-f] thieno [3, 2-b] thiophene (DNTT) and its didecyl and diphenyl derivatives. Organic Electronics. 2016 Aug 1;35:33-40.
28. Kim MJ, Lee TI, Lee C, Shin EJ, Kim S, Jeong J, Hwang WS, Im SG, Cho BJ. Performance enhancement of p-type organic thin-film transistors by surface modification of hybrid dielectrics. Organic Electronics. 2021 Sep 1;96:106250.

29. Sarkar A. Device simulation using Silvaco ATLAS tool. InTechnology computer aided design 2018 Sep 3 (pp. 203-252). CRC Press.
30. Taouririt TE, Meftah A, Sengouga N. Numerical Simulation and Optimization of An a-ITZO TFT Based on a Bi-Layer Gate Dielectrics. Journal of Electronic Materials. 2019 Feb 15;48:1018-30.
31. Zaki T, Scheinert S, Hörselmann I, Rödel R, Letzkus F, Richter H, Zschieschang U, Klauk H, Burghartz JN. Accurate capacitance modeling and characterization of organic thin-film transistors. IEEE Transactions on Electron Devices. 2013 Dec 5;61(1):98-104.
32. TEJA KARRI BR, Gupta N. Hybrid bilayer gate dielectric-based organic thin film transistors. Bulletin of Materials Science. 2019 Feb;42(1):2.
33. Veres J, Ogier SD, Leeming SW, Cupertino DC, Mohialdin Khaffaf S. Low-k insulators as the choice of dielectrics in organic field-effect transistors. Advanced Functional Materials. 2003 Mar 4;13(3):199-204.
34. Alghamdi NA. Study and Analysis of Simple and Precise of Contact Resistance Single-Transistor Extracting Method for Accurate Analytical Modeling of OTFTs Current-Voltage Characteristics: Application to Different Organic Semiconductors. Crystals. 2021 Nov 24;11(12):1448.
35. Park J, Kang JM, Kim DW, Choi JS. Contact resistance variation in top-contact organic thin-film transistors with the deposition rate of Au source/drain electrodes. Thin Solid Films. 2010 Sep 1;518(22):6232-5.
36. Ye X, Zhao X, Wang S, Wei Z, Lv G, Yang Y, Tong Y, Tang Q, Liu Y. Blurred electrode for low contact resistance in coplanar organic transistors. ACS nano. 2020 Dec 18;15(1):1155-66.
37. Deman AL, Tardy JJ. PMMA–Ta2O5 bilayer gate dielectric for low operating voltage organic FETs. Organic Electronics. 2005 Apr 1;6(2):78-84.
38. Choi JM, Hwang DK, Jeong SH, Park JH, Kim E, Kim JH, Im S. Polymer/AlO x bilayer dielectrics for low-voltage organic thin-film transistors. Journal of the Electrochemical Society. 2007 Mar 12;154(5):H331.
39. Yoo S, Kim YH, Ka JW, Kim YS, Yi MH, Jang KS. Polyimide/polyvinyl alcohol bilayer gate insulator for low-voltage organic thin-film transistors. Organic Electronics. 2015 Aug 1;23:213-8.
