# Supplementary material for: Investigation of gate dielectric interface on contact resistance of short channel organic thin film transistors (OTFT)
Source: PLoS One. 2025 Sep 11;20(9):e0326929. doi: 10.1371/journal.pone.0326929 (PMC12425321; doi:10.1371/journal.pone.0326929)
Supplement: Attachment — Submitted filename: Response - to-Reviewers.docx [file pone.0326929.s003.docx]

**Original Manuscript Number:** PONE-D-24-59959

**Original Article Title: “Investigation of Gate Dielectric Interface on Contact Resistance of Short Channel Organic Thin Film Transistors (OTFT)”**

**To:** PLOS ONE Editor

**Re:** Response to reviewers

Dear Editor,

Thank you for allowing a resubmission of revised manuscript and giving us an opportunity to address the reviewers’ comments.

We are uploading (a) our point-by-point response to the comments (below) (response to reviewers, under “Author’s Response Files*”*), (b) an updated manuscript with yellow highlighting indicating changes (as “Revised Manuscript with Track Changes*”*), and (c) a clean updated manuscript without highlights (“Manuscript”*).*

Best regards,

Dr. M. Durga Prakash

**Reviewer#1, Concern # 1:** All the analysis are based on the simulation, while the simulation parameters are not presented, especially for interface trap density of different thickness, which is critical for reliability assessment. More detailed analysis and related reference should be provide. What's more, the bulk trap in Al₂O₃ is also a great concern. The analysis should be provided.

**Author response:** We appreciate the reviewer’s emphasis on the importance of clearly presenting simulation parameters and trap-related analysis, particularly in assessing OTFT reliability.

**Author action:** we have significantly revised the manuscript to include a comprehensive summary of simulation parameters (Table 1), including material properties, mobility models, and geometric details used in the Silvaco Atlas simulations.

**Table 1**. Summary of structure dimensions and material parameters used in simulation

| **Classification** | **Parameters** | **Values /units** |
| --- | --- | --- |
| Physical Dimensions | Channel Length (L_ch_) | 1 µm |
|  | Width (W) | 200 µm |
|  | Thickness of DNTT (T_osc_) | 25 nm |
|  | Thickness of single dielectric Al_2_O_3_, PVP (T_die_) | Fixed at 12 nm |
|  | Thickness of hybrid dielectric Al₂O₃ / PVP (T_die_) | Varied for different compositions |
|  | Thickness of electrodes(T_sd_,T_g_) | 30 nm |
| Materials  Organic SC-D NTT | Bandgap (eg) | 3.38 [34] |
|  | Permittivity (ε) | 3 |
|  | Electron Affinity (χ_e_) | 1.81 |
|  | Effective DOS of LUMO (N_c_) | 1e20 cm^-3^ |
|  | Effective DOS of HOMO(N_v_) | 1e20 cm^-3^ |
|  | Doping Concentration | 1x10^15^ cm^-3^ |
| Al_2_O_3_ | Dielectric Constant | 9.3 [35] |
|  | Bulk trap density | 1x10^14^ cm^-3^ |
| PVP | Dielectric Constant | 3.8 |
| Work function | Gold (Au) | 5.0 eV |
|  | Aluminium (Al) | 4.28 eV |
| PF Mobility Parameters | deltaep.pfmob | 1.792e-1 eV |
|  | betap.pfmob | 7.785e5 eV(cm/V)^1/2^ |
|  | Temperature (K) | 300K |

- Regarding interface trap density and about bulk trap states in Al_2_O_3_, we have added a detailed explanation in the Simulation Methodology section on how N_it_ was extracted from the subthreshold slope (SS) using Eq. (16). We further tabulated the extracted N_it_ values for different dielectric configurations in Table 2 and discussed their correlation with dielectric thickness and performance metrics. These findings demonstrate how hybrid dielectric structures (especially PVP-rich stacks) help suppress interface trap density and improve mobility as follows

Table 1 enumerates the material input parameters, including device dimensions, dielectric properties, and mobility models used in the TCAD simulations for OTFTs with hybrid and single-layer dielectrics.

To account for the effect of trapped charges, Poisson’s equation was modified to incorporate spatial charge contributions from ionized traps:

$div(\varepsilon\nabla\Psi)=q(n-p-N_{tD}^{+}+N_{A}^{-})-Q_{T}$	(10)

where $Q_{T}=q\left( N_{tD}^{+} +N_{tA}^{-} \right)$ represents the total trapped charge density

Here, $N_{tD}^{+}=trap density \times F_{tD}$ and $N_{tA}^{-}=trap density \times F_{\begin{aligned} tA \\ \end{aligned}}$

Here, $N_{tD}^{+}$ and $N_{tA}^{-}$ are the ionized donor- and acceptor-like trap densities respectively and $F_{tD}$ and $F_{tA}$ are their respective ionization probabilities. Organic semiconductors inherently, contain a tail and deep -level traps within the band gap, which are described using both exponential (tail) and Gaussian (deep) density-of-states (DOS) functions.

The equations that describe these terms are as follows

$$g\left( E \right)=g_{TA}\left( E \right)+g_{TD}\left( E \right)+g_{GA}\left( E \right)+g_{GD}\left( E \right) (11)$$

where the tail and deep level bands are modeled with an exponential and Gaussian distributions, respectively, as:

$$g_{TA}\left( E \right)=N_{TA}exp\left[ \frac{E - E_{c}}{W_{TA}} \right] (12)$$

$g_{TD}\left( E \right)=N_{TD}exp\left[ \frac{E_{V} - E}{W_{TD}} \right] (13)$

$g_{GA}\left( E \right)=N_{GA}exp\left[ -\left[ \frac{E_{GA}-E}{W_{GA}} \right]^{2} \right] (14)$

$g_{GD}\left( E \right)=N_{GD}exp\left[ -\left[ \frac{E-E_{GD}}{W_{GD}} \right]^{2} \right] (15)$

Here, E is the trap energy, E_C_ is the conduction band energy, E_V_ is the valence band energy and the subscripts (T, G, A, D) stand for tail, Gaussian (deep level), acceptor and donor states respectively.

The quantities N_TA_, N_TD_, N_GA_, N_GD_ and W_TA_, W_TD_, W_GA_, W_GD_ are the fitting parameters of TRAP models. In our simulations, both interface and bulk trap effects were incorporated using Silvaco Atlas INTDEFECTS and INTERFACE models to accurately capture semiconductor-dielectric and semiconductor-contact interfacial behaviors. Specifically, we implemented a Gaussian distribution of bulk traps in the Al_2_O_3_layer with a peak trap density of 1×10^14^ cm^−3^, centered at 0.6 eV below the conduction band edge [32][33]. This bulk trap model significantly influenced gate leakage and off-state current in single-layer Al_2_O_3_ devices. To quantify interface trap density (N_it_), we extracted values from the subthreshold slope (SS) of the transfer characteristics using the established relation.

$$N_{it}=\left( \frac{SS}{ln10}\frac{q}{KT}-1 \right)\frac{C_{ox}}{q} (16)$$

Where C_ox_ is the gate oxide capacitance per unit area, and SS is extracted from the logarithmic plot of drain current versus gate voltage. Extracted N_it_ values, summarized in Table 2, reveal a strong dependence on dielectric stack configuration, particularly for the Al_2_O_3_/PVP hybrid system. Notably, an optimized PVP thickness (e.g., 3.6–4.2 nm) substantially suppresses interface trap density, improving carrier mobility and subthreshold performance. It is important to note that N_it_ values are sensitive to process-dependent factors, including surface roughness, polymer cross-linking, and metal-oxide interface quality.

[32] Shi, Yuanyuan, et al. "Investigation of bulk traps by conductance method in the deep depletion region of the Al 2 O 3/GaN MOS device." *Nanoscale research letters* 12 (2017): 1-6.

[33] Deng, Kexin, et al. "Insight into the suppression mechanism of bulk traps in Al_2_O_3_ gate dielectric and its effect on threshold voltage instability in Al_2_O_3_/AlGaN/GaN metal-oxide-semiconductor high electron mobility transistors." *Applied Surface Science* 638 (2023): 158000.

These revisions ensure that both interface and bulk defect contributions are clearly modeled, quantified, and linked to the device's electrical performance. We thank the reviewer for prompting us to clarify and expand this critical aspect of our simulation framework.

**Reviewer#1, Concern # 2:** Why hybrid dielectric layers is selected as Al_2_O_3_/PVP, rather than other stacks, such as HfO2/PMMA, should be clarified.

**Author response:** We appreciate the reviewer’s suggestion to clarify the rationale for selecting the Al_2_O_3_/PVP hybrid dielectric system. Our choice was guided by several considerations:

1. **Dielectric Constant Complementarity**: Al_2_O_3_ offers a relatively high dielectric constant (~9.3), which allows for low-voltage operation, while PVP has a low-κ (~4.7) value, which is effective in suppressing gate leakage and minimizing interface traps.
2. **Interface Compatibility with DNTT**: PVP forms a smooth organic–organic interface with the DNTT semiconductor, facilitating molecular ordering and reducing charge scattering. This is particularly advantageous compared to PMMA, which is less favourable in forming high-quality interfaces with DNTT.
3. **Thermal and Mechanical Stability**: PVP films can be cross-linked thermally, offering stable and uniform thin layers. This enables the realization of sub-5 nm interfacial layers critical for hybrid stack tuning.
4. **Literature Precedence and Availability**: Al_2_O_3_ and PVP are both widely used and well-characterized in OTFT research. Prior studies (e.g., Kraft et al. [27]) have demonstrated the compatibility of this stack with short-channel, high-mobility OTFTs.

While HfO_2_/PMMA is a viable alternative, our selection of Al_2_O_3_/PVP was based on achieving a balance between process feasibility, device performance, and interface quality specific to our DNTT-based architecture. We have now clarified this material rationale in the revised manuscript.

er.

**Author action:** We have updated the manuscript to include the importance of Al_2_O_3_/PVP hybrid dielectric stack in the introduction section as follows.

In this context, we chose Al_2_O_3_/PVP as the hybrid dielectric stack, due to their complementary material properties and proven compatibility with organic semiconductors. Al_2_O_3_ offers a high dielectric constant (~9), excellent thermal stability, and favorable electrical performance at low voltages. Meanwhile, PVP contributes hydrophobicity, low interface trap density, mechanical flexibility, and biocompatibility. This specific combination was selected over other stacks because (i) Al_2_O_3_ provides better interfacial quality and lower trap density compared to high -K dielectric materials which often introduces instability in OTFTs, and (ii) PVP exhibits better uniform film formation and fewer residual polar groups than organic dielectrics, which improves the semiconductor–dielectric interface. These advantages make Al_2_O_3_/PVP a robust choice for hybrid dielectric engineering in high performance OTFTs.

**Reviewer#1, Concern # 3:**  This article is primarily about simulation, but there is very little detailed explanation of the specific microscopic mechanisms, especially how the gate stack affects the contact mechanism, which needs to be explained in detail.

**Author response:** We sincerely thank the reviewer for highlighting the need for a more detailed microscopic explanation of how the gate dielectric stack impacts the contact resistance mechanism.

**Author action:** We have significantly expanded the discussion section, to clarify the electrostatic and interfacial mechanisms underlying this effect, supported by literature references and energy band considerations.

The efficiency of OTFTs is significantly influenced by the properties of the gate dielectric, particularly at the dielectric-semiconductor interface, which directly affects the injection and transport of charge carriers. To enhance the overall performance and stability of the device, we adopted a bilayer dielectric structure composed of a high-k inorganic layer (Al_2_O_3_) and an organic interfacial layer (PVP). This hybrid configuration exploits the advantages of both materials: the high dielectric constant of Al_2_O_3_ (κ ≈ 9.3) increases gate capacitance and promotes low-voltage operation, while the polymeric PVP layer offers a smooth surface morphology and a lower density of interface traps that otherwise impede carrier mobility and degrade contact behavior.

The gate dielectric stack has a critically affects energy-level alignment and charge injection at the source/drain contact interface. In single Al_2_O_3_ dielectric OTFT illustrated in Fig 10(a), direct contact between Al_2_O_3_ and the DNTT semiconductor often introduces a significant density of interface traps, arising from lattice mismatch, surface roughness, and hydroxyl-related dipoles at the oxide surface. These traps serve as scattering centers and contribute to Fermi level pinning, which inhibits efficient carrier accumulation and widens the Schottky barrier at the metal–semiconductor junction, thereby increasing contact resistance.

In contrast, the hybrid Al_2_O_3_ /PVP dielectric stack as shown in Fig 10(b), introduces a thin PVP layer at the dielectric-semiconductor interface. Due to its organic nature and surface energy compatibility with DNTT, PVP minimizes interfacial trap density and promotes improved molecular ordering at the interface. As a result, the trap-induced Fermi level pinning at the source/drain contact edges is reduced, enabling more efficient carrier injection. Consequently, the effective Schottky barrier width is reduced, facilitating thermionic emission or tunneling-based carrier injection and lowering contact resistance. Furthermore, the reduced interfacial trap density allows a greater portion of the gate potential to modulate the channel, thereby improving the subthreshold swing and effective carrier mobility.

This interfacial mechanism is illustrated in Fig 10. In Figs 10a, the single Al_2_O_3_ introduces more interface traps, distorting the band profile and impeding carrier injection. In contrast, Fig. 10b shows that the hybrid dielectric enables smoother band alignment and fewer trap states, enhancing charge injection and lowering contact resistance. Thus, the gate stack not only governs electrostatic control over the channel but also critically influences the contact injection process [41][42].

[41] Kim, Kang Dae, and Chung Kun Song. "Low-voltage organic thin-film transistors using a hybrid gate dielectric consisting of aluminum oxide and poly (vinyl phenol)." *Japanese journal of applied physics* 49.11R (2010): 111603.

[42] Soltani, B., M. Babaeipour, and A. Bahari. "Studying electrical characteristics of Al 2 O 3/PVP nano-hybrid composites as OFET gate dielectric." *Journal of Materials Science: Materials in Electronics* 28 (2017): 4378-4387

**Reviewer#1, Concern # 4** The simulation assumes a "trap-free interface" at the Al_2_O_3_ /PVP boundary. This simplification may overlook real world interfacial defects, which are critical for OTFT reliability.

**Author response:** We thank the reviewer for pointing out this inconsistency. We acknowledge that an earlier version of the manuscript may have caused confusion by suggesting that interface defects were not considered. To clarify:

- Our simulation explicitly includes defects at the semiconductor–dielectric interface, particularly at the DNTT/PVP and DNTT/Al₂O₃ boundaries.
- Interface trap density (N_it_) was extracted from the subthreshold slope using Eq. (16) and implemented using Silvaco’s INTERFACE and INTTRAP models.
- These interface traps were essential for accurately simulating hysteresis behavior, subthreshold swing, and leakage current in the OTFTs.
- We have revised the manuscript accordingly to ensure consistency and have removed the misleading wording suggesting that the interface was considered trap-free.

**Author action**: we have updated the manuscript, included interface trap modeling in our simulation framework. Specifically:

To evaluate the effect of interface traps on device performance, we extracted the interface trap density (N_it_) from the subthreshold slope (SS) of the simulated transfer characteristics using the following relation

$$N_{\mathrm{it}}=\left( \frac{\mathrm{SS}}{ln10}\frac{q}{\mathrm{KT}}-1 \right)\frac{C_{\mathrm{ox}}}{q} (16)$$

Where C_ox_ is the gate oxide per unit area, and SS is extracted from the logarithmic plot of drain current versus gate voltage. These extracted N_it_ values were then incorporated back into the simulation using Silvaco’s INTERFACE module. This modeling approach allowed us to assess the quantitative impact of interfacial traps on key parameters such as threshold voltage, mobility, and hysteresis behavior in both single and hybrid dielectric OTFT configurations.

**Reviewer#1, Concern # 5** It seems that only a very thin layer of low-k dielectric is needed to improve the interface characteristics between the high-k dielectric and OTFT. Are there any relevant methods in actual processes to control the thickness of the low-k dielectric?

**Author response:** We thank the reviewer for this important observation. Yes, in practical fabrication processes, there are well-established methods to precisely control the thickness of ultra-thin low-k polymer dielectrics such as poly(4-vinylphenol) (PVP)

**Author action**: We have revised the manuscript by including relevant fabrication techniques in the simulation section, supported by appropriate references.

Although such ultra-thin polymer layers are challenging to fabricate, they are achievable using controlled spin-coating techniques, where the film thickness is modulated through solution concentration and spin speed. Fan et al. [29] demonstrated the successful deposition of sub-5 nm PVP layers atop a PVA layer, which improved organic thin-film transistor performance by enhancing surface morphology and enabling better molecular ordering of the semiconductor layer. Additionally, post-deposition thermal cross-linking improves film uniformity and mechanical stability. These findings affirm the experimental feasibility of integrating ultra-thin PVP interfacial layers in high-performance OTFT devices, consistent with our simulation assumptions.

[29] Fan, Ching-Lin, et al. "Performance enhancement of pentacene-based organic thin-film transistors using a high-K PVA/low-K PVP bilayer as the gate insulator." *Polymers* 13.22 (2021): 3941

**Reviewer#2, Concern # 1** The manuscript presents a model of 2d transistors, where the appropriate set of differential equations are solved, my question here is how does the simulation deals with the boundary conditions.

**Author response:** We thank the reviewer for pointing out the need to clarify boundary conditions in our 2D simulation framework. In our 2D Silvaco Atlas simulation, the boundary conditions are handled as follows:

- **Electrode Boundaries (Source/Drain/Gate):** The source and drain contacts were defined by assigning specific metal work-functions to represent Schottky-type contacts. The resulting contact barriers were determined by the difference between the electrode work-function and the HOMO level of the organic semiconductor (DNTT). This approach allows the simulator to self-consistently calculate the injection barrier and current crowding effects at the contacts.
- **Gate Contact:** The gate boundary was treated as an ideal metal with a fixed work-function, and a voltage sweep was applied during transfer and C-V characteristic simulations.
- **Neumann (Zero-Flux) Boundary Conditions:** At the lateral and bottom boundaries of the simulation domain (i.e., non-contact edges), default Neumann boundary conditions were applied to ensure zero normal current flow across insulating regions.
- **Interface Modeling:** The dielectric–semiconductor and semiconductor–metal interfaces were treated with Silvaco's INTDEFECTS and INTERFACE models, incorporating both interface and bulk traps as described in the revised simulation section.

This setup allowed us to realistically simulate charge injection, accumulation, and transport under various gate and drain biases. We have included a clarification of the boundary condition handling in the Simulation Methodology section for completeness.

**Author action:** We have revised the manuscript by including the boundary conditions in simulation approach section as follows

Boundary conditions were assigned as follows: the source and drain contacts were treated as Schottky-type boundaries with defined work-functions, the gate contact was modeled as an ideal metal with Dirichlet potential. All non-contact edges were treated as Neumann (zero-flux) boundaries. Interface continuity and trap behavior were incorporated using the INTERFACE and INTDEFECTS models.

**Reviewer#2, Concern # 2** At some point it establish that they does not consider defects at the interface of the semiconductor-dielectric interface, however some of the parameters like the hysteresis curve for example clearly depends of the density of these defects.

**Author response:** We thank the reviewer for pointing out this inconsistency. We acknowledge that an earlier version of the manuscript may have caused confusion by suggesting that interface defects were not considered. To clarify:

- Our simulation explicitly includes defects at the semiconductor–dielectric interface, particularly at the DNTT/PVP and DNTT/Al₂O₃ boundaries.
- Interface trap density (N_it_) was extracted from the subthreshold slope using Eq. (16) and implemented using Silvaco’s INTERFACE and INTTRAP models.
- These interface traps were essential for accurately simulating hysteresis behavior, subthreshold swing, and leakage current in the OTFTs.
- We have revised the manuscript accordingly to ensure consistency and have removed the misleading wording suggesting that the interface was considered trap-free.

**Author action**: We have updated the manuscript by how we evaluated the N_it_

To evaluate the effect of interface traps on device performance, we extracted the interface trap density (N_it_) from the subthreshold slope (SS) of the simulated transfer characteristics using the following relation

$$N_{\mathrm{it}}=\left( \frac{\mathrm{SS}}{ln10}\frac{q}{\mathrm{KT}}-1 \right)\frac{C_{\mathrm{ox}}}{q} (16)$$

Where C_ox_ is the gate oxide per unit area, and SS is extracted from the logarithmic plot of drain current versus gate voltage. These extracted N_it_ values were then incorporated back into the simulation using Silvaco’s INTERFACE module. This modeling approach allowed us to assess the quantitative impact of interfacial traps on key parameters such as threshold voltage, mobility, and hysteresis behavior in both single and hybrid dielectric OTFT configurations

**Reviewer#2, Concern # 3(a)** The contact resistance in given in Ohms instead of Ohm-cm^2, the drain to source is also given the currents instead of a density of currents as i expected from a 2d model.

**Author response:** We thank the reviewer for highlighting this important distinction in unit conventions for 2D device simulations.

**Author action**: we have revised the reported contact resistance and now express it in terms of area-specific contact resistance (Ω·cm²), which is more appropriate for a 2D simulation framework.

**Reviewer#2, Concern # 3(b)** Figure 2 and 12 the numbers are so tiny that its difficult to read.

**Author response:** We appreciate the reviewer’s observation regarding the readability of Figures 2 and 12.

**Author action:** We have updated the figures in the manuscript with higher resolution versions and improved the axis labels for better clarity. This includes adjustments to font size, scaling, and axis labelling to ensure that the data is more accessible and easier to interpret.

**Reviewer#2, Concern # 4** There are several parameters just before the results that are not clearly explained what they are.

**Author response:** We thank the reviewer for pointing out the need to improve clarity in the presentation of parameters introduced before the Results section.

**Author action**: In the revised manuscript, we have carefully reviewed this section and provided clear definitions and physical interpretations for all simulation and material parameters, including trap-related DOS terms (e.g., N_GA_,W_GA_) and mobility model constants. Each symbol is now described at first mention, and units are provided where applicable. These clarifications improve the accessibility of the technical content and ensure the reader can follow the modeling assumptions and their relevance to the simulation outputs.

**Reviewer#2, Concern # 5** The model does not consider defects on the interface Al_2_O_3_-PVP, could you please provide until what density of defects is still more convenient to use the hybrid layer instead of a single one.

**Author response:** We appreciate the reviewer’s careful distinction between different interfaces in the device. In our simulations, we have incorporated detailed trap modeling at the semiconductor–dielectric interface (DNTT/PVP or DNTT/ Al_2_O_3_) as well as within the bulk Al_2_O_3_ layer. However, the Al_2_O_3_–PVP buried interface within the hybrid dielectric was assumed to be electrically passive in our model, i.e., no traps were assigned at that internal dielectric-dielectric interface.

We agree that in real devices, dipoles, roughness, or chemical mismatches at the Al_2_O_3_–PVP interface could introduce trap states or fixed charges. While we did not simulate a parametric sweep of such defect densities, based on our experience with interface trap modeling at other boundaries, we estimate that if the Al_2_O_3_–PVP interface were to exhibit trap densities exceeding ~10¹² cm⁻²·eV⁻¹, it could degrade gate modulation and reduce the advantages of the hybrid dielectric configuration—especially in short-channel devices.

**Author action**: We have revised manuscript by including in results and discussion section

While the Al_2_O_3_–PVP interface was assumed ideal in this study, moderate interfacial defect densities could influence electric field distribution. Future simulations should examine their impact to further validate the reliability of this hybrid configuration.

**Reviewer#2, Concern # 6** On the results and discussion firs paragraph the density of trap defect has a typo should be order of 10^11

**Author response:** We thank the reviewer for catching this typographical error.

**Author action:** We have revised the manuscript to include the corrected order of magnitude for the interface trap density in the first paragraph of the Results and Discussion section should indeed be 10^11^ cm^−2^ not 10^-11^.

**Reviewer#2, Concern # 7** - In the manuscript argument that the leakage current and reduce mobility is affected by a random distribution of dipoles that disorganize the energy landscape that make sense but explain how this is included on your model.

**Author response**: We thank the reviewer for this thoughtful observation. In the manuscript, the reference to random dipoles at the Al_2_O_3_ interface was intended as a physical explanation for the observed degradation in mobility and increased leakage current in devices with high-κ dielectrics. These dipoles introduce energetic disorder that disrupts charge transport, as discussed by Veres et al. [33].

While our simulation does not explicitly model spatially distributed dipoles, we account for their effects indirectly through the density-of-states (DOS) model implemented in Silvaco Atlas. Specifically, we introduced broadened exponential tail states and Gaussian deep-level traps in the DOS, which reflect the energetic disorder typically associated with dipolar fluctuations. These DOS parameters were calibrated to reproduce experimental characteristics, including subthreshold slope and field-effect mobility.

We have clarified this point in the revised Results and Discussion section, and emphasized that dipolar disorder is incorporated into the simulation via its impact on trap energetics and transport behavior, rather than through discrete dipole-field modeling.

**Author action:** We have revised the manuscript “According to Veres et al., these dipoles can disorganize the energy landscape, leading to reduced field-effect mobility in OTFTs [35]. Although we did not explicitly model random dipoles as discrete entities, their effects were incorporated indirectly through broadened exponential tail states and Gaussian deep traps in the DOS model. These features effectively capture the impact of energetic disorder on mobility and leakage behavior, consistent with experimental observations.

**Couple More Responses for Other Comments on the PDF Attached.**

**Reviewer#2, Concern #1** could you explain how you treat the boundaries of your simulation cell.

**Author response:** We thank the reviewer for highlighting the need to clarify the boundary treatment in our simulation. In the revised manuscript, we have added explicit details regarding the boundary conditions used in the 2D Silvaco Atlas simulations.

Specifically:

- The source and drain electrodes were defined as Schottky-type contacts by assigning metal workfunctions, allowing the simulator to self-consistently calculate injection barriers based on the offset with the HOMO level of the DNTT semiconductor.
- The gate contact was modeled as an ideal metal, with the applied gate voltage defined directly as a Dirichlet boundary condition.
- Lateral and bottom boundaries of the simulation domain were treated using Neumann (zero-flux) boundary conditions, ensuring no artificial current injection or loss through non-contact surfaces.
- At dielectric–semiconductor interfaces, continuity of electric displacement and carrier concentration was enforced using the INTERFACE and INTDEFECTS models, which also incorporated trap-assisted transport.

**Author action**: These boundary conditions ensure physical consistency and numerical convergence within the simulation domain. We have incorporated this clarification into the Simulation Methodology section of the revised manuscript.:

“Boundary conditions were assigned as follows: the source and drain contacts were treated as Schottky-type boundaries with defined workfunctions; the gate contact was modeled as an ideal metal with Dirichlet potential. All non-contact edges were treated as Neumann (zero-flux) boundaries. Interface continuity and trap behavior were incorporated using the INTERFACE and INTDEFECTS models.”

**Reviewer#2, Concern # 2** If your are using a trap-free interface model what is the necessity of using this equation?

**Author response:** We thank the reviewer for pointing out this inconsistency. However, as the analysis progressed, especially in evaluating subthreshold swing and hysteresis, we incorporated interface traps into the model to more accurately reflect experimental behavior and reliability factors.

The Equation (16 ) to extract interface trap density (N_it_) is part of this extended modeling phase. The extracted Nit values were subsequently included in the simulation using Silvaco’s INTERFACE and INTDEFECTS modules. Thus, the equation is not used in the idealized trap-free case, but rather in the realistic device model used for final analysis and comparison with fabricated devices.

We have clarified this progression in the manuscript and adjusted the simulation description accordingly to reflect the two-stage modeling approach (initial trap-free → trap-included).

.

**Author action:** We have revised the manuscript To evaluate the effect of interface traps on device performance, we extracted the interface trap density (N_it_) from the subthreshold slope (SS) of the simulated transfer characteristics using the following relation

$$N_{\mathrm{it}}=\left( \frac{\mathrm{SS}}{ln10}\frac{q}{\mathrm{KT}}-1 \right)\frac{C_{\mathrm{ox}}}{q} (16)$$

Where C_ox_ is the gate oxide per unit area, and SS is extracted from the logarithmic plot of drain current versus gate voltage. These extracted N_it_ values were then incorporated back into the simulation using Silvaco’s INTERFACE module. This modeling approach allowed us to assess the quantitative impact of interfacial traps on key parameters such as threshold voltage, mobility, and hysteresis behavior in both single and hybrid dielectric OTFT configurations

**Reviewer#2, Concern # 3** what is Cox, the capacitance of the Al203-PVP ?

**Author response:** We thank the reviewer for pointing out the need to clarify the definition of Cox in Equation (9). For hybrid dielectric configurations (Al_2_O_3_/PVP), C_ox_ was calculated as the effective series capacitance per unit area of the bilayer stack, based on the thickness and dielectric constants of each layer:

$$\frac{1}{C_{OX}}=\frac{1}{C_{Al2O3}}+\frac{1}{C_{PVP}} (2)$$

**Author action:** We have revised the manuscript to include the C_ox_ equation

For hybrid dielectric configurations (Al_2_O_3_/PVP), Cox was calculated as the effective series capacitance of the two dielectric layers

$$\frac{1}{C_{OX}}=\frac{1}{C_{Al2O3}}+\frac{1}{C_{PVP}} (2)$$

where C_Al2O3_ and C_PVP_ are the individual capacitances derived from their respective dielectric constants and physical thicknesses. For single-layer devices, Cox was computed using the standard parallel-plate formula.

**Reviewer#2, Concern # 4** the density of state parameters was adjusted to W_TA_ = 0.013, W_TD_ = 0.12, N_GA_ = 1.2x10^14^, N_GD_ = 2x10^16^, E_GA_ = 1.2, E_GD_ = 2.9, and W_GA_ = 0.05. What are this values, please explain.

**Author response:** We appreciate the reviewer’s request for clarification. The parameters listed correspond to the density-of-states (DOS) model used to represent the distribution of trap states within the organic semiconductor (DNTT). Specifically:

W_TA_,W_TD_ represent the energy widths of exponential tail states for acceptor and donor traps, respectively (in eV).

N_GA_,N_GD_ are the peak densities of deep Gaussian acceptor and donor traps (in cm⁻³).

E_GA_,E_GD_ indicate the energy positions of these Gaussian peaks, typically measured from the valence band edge (in eV).

W_GA_,W_GD_ define the widths (standard deviations) of the Gaussian distributions (in eV).
These parameters were calibrated to match experimental data and characterize the energetic disorder in the organic semiconductor (DNTT).

**Author action:** We have revised the manuscript as follows

The subscripts (T, G, A, D) stand for tail, Gaussian (deep level), acceptor and donor states respectively.

The quantities N_TA_, N_TD_, N_GA_, N_GD_ and W_TA_, W_TD_, W_GA_, W_GD_ are the fitting parameters of TRAP models.

**Reviewer#2, Concern # 5** which mobility are you referencing here? the field dependent, the saturation or something else, and it might be obvious but please make clear of you get the leakage current from your simulation.

**Author response:** We thank the reviewer for this valuable clarification request. The reported mobility value of 3.86 cm²/V·s corresponds to the effective field-effect mobility, extracted from the linear region of the transfer characteristics at low drain voltage (V_DS_=-0.1V) using the standard transconductance method Eq. (6)

$$\mu_{\mathrm{eff}}=\frac{Lg_{m}}{wC_{\mathrm{ox}}}\times\frac{1}{V_{\mathrm{DS}}} (6)$$

This effective mobility was calculated from the simulated transfer curve. We have now explicitly stated this in the Results section for clarity.

Regarding the leakage current density (10^-6^ A/cm²), this value was also obtained from our Silvaco Atlas simulation under off-state bias conditions (V_GS_=0 V,V_DS_=−1.5). It reflects the gate-induced leakage through the bulk and interfacial trap pathways in the Al_2_O_3_ dielectric. We have revised the manuscript text to clearly indicate that both the mobility and leakage current were extracted from simulation outputs.

**Author action:** We have revised the manuscript as However, despite this low threshold voltage, the Al_2_O_3_-based device shows reduced effective field-effect mobility (3.86 cm²/V·s), calculated from the linear region of the simulated transfer curve using the transconductance method, and an elevated leakage current density (~10^-6^ A/cm²), obtained from the simulated off-state condition at (V_GS_=0 V,V_DS_=−1.5V)

**Reviewer#2, Concern # 6** The explanation of why the Al_2_O_3_ interfaces have a higher leakage current make sense but my question is how you are able to model this issue if you are not considering the effect of random dipoles on the aluminium oxide,

**Author response:** We appreciate the reviewer’s insightful follow-up. As noted, we did not explicitly model random dipole distributions at the Al₂O₃ interface as localized physical entities in the simulation. Instead, we accounted for their electrical effects indirectly through a calibrated density of states (DOS) model and interface trap profile.

Specifically, the increased leakage current observed in Al₂O₃-based devices was reproduced in simulation by incorporating a higher interface trap density and a Gaussian-distributed bulk trap profile within the Al₂O₃ layer (centered at 0.6 eV below the conduction band). These features capture the effect of energetic disorder caused by dipolar fluctuations, as described in literature [e.g., Veres et al.].

Thus, while we did not assign spatially varying dipole fields, the resulting trap-enhanced carrier injection and off-state conduction are embedded in our model through trap-assisted leakage mechanisms. We have clarified this point in the revised manuscript to ensure consistency between our physical explanation and simulation methodology.

**Author action:**  Although we did not explicitly model spatially distributed dipoles at the Al₂O₃ interface, their influence was indirectly captured through a higher interface trap density and a Gaussian-distributed bulk trap profile within the Al₂O₃ layer, centered at 0.6 eV below the conduction band. This approach allowed the simulation to reflect the leakage-enhancing effects of dipolar disorder through trap-assisted conduction mechanisms, consistent with observed trends and literature reports such as Veres et al.

**Reviewer#2, Concern # 7** Your model does not consider the possibility of defects on the interface Al203-PVP, that could lead to decrease the electric field and therefore impact on the perfomace of the transistor, could you estimate if a moderate density of defects on the Al_2_0_3_ -pvp interfaces could change your conclusions?

**Author response**: We thank the reviewer for this important observation. In our simulation, the Al₂O₃–PVP interface was assumed to be ideal and free of defect states, focusing on the dominant trap contributions at the semiconductor–dielectric (PVP/DNTT) interface and within the Al₂O₃ bulk. We agree that, in reality, the buried Al₂O₃–PVP interface could exhibit dipolar disorder, fixed charges, or interface roughness, which may alter the local electric field and affect device performance.

While we did not explicitly model this interface in the current study, we expect that a moderate density of interfacial defects could lead to some reduction in field coupling and marginal degradation of mobility or threshold control. However, based on prior literature and the dominant role of the semiconductor-facing interface, we anticipate that the overall advantage of the hybrid dielectric structure would remain qualitatively valid.

**Author action:** We have updated the manuscript as follows

While the Al₂O₃–PVP interface was assumed ideal in this study, moderate interfacial defect densities could influence electric field distribution. Future simulations should examine their impact to further validate the reliability of this hybrid configuration.

**Reviewer#2, Concern # 8** did you consider defect at the interfaces at all?

**Author response:** We thank the reviewer for this important clarification. Yes, our model explicitly considered defects at the semiconductor–dielectric interface (DNTT/PVP or DNTT/Al₂O₃), which were included via the INTERFACE and INTDEFECTS modules in Silvaco Atlas. The interface trap density (Nit) values were extracted from the subthreshold slope (SS) using Equation (16) and incorporated into the simulation to model their impact on mobility and subthreshold characteristics.

**Author action:** This mobility enhancement is supported by simulation using interface trap models at the semiconductor–dielectric (PVP/DNTT) interface.

**Reviewer#2, Concern # 9** on your model the bandgap of the pvp is higher than the Al203, this is not correct

**Author response:** We thank the reviewer for catching this important inconsistency in Figure 10(b). The band alignment in the diagram incorrectly suggests that the polymer PVP has a larger bandgap than Al_2_O_3_, which is not physically accurate. Al_2_O_3_, a high-κ metal oxide, has a reported bandgap in the range of 7–9 eV, while PVP has a smaller bandgap around 4–5 eV. We have revised the energy band diagram in Figure 10(a) to reflect the correct relative bandgaps and improve the accuracy of the interfacial energy level representation.

**Author action:** The fig.10(b) has been changed and updated in the manuscript

**Fig 10.** Schematic band energy diagram of the dielectric-semiconductor interface in a DNTT based OTFT with (a) single Al_2_O_3_ dielectric, (b) Al_2_O_3_/PVP gate hybrid dielectric

**Reviewer#2, Concern # 10** please explain how this time dependence phenomena is included on your model.

**Author response:** We thank the reviewer for raising this important point regarding the time-dependent phenomena reflected in the hysteresis behavior. In our simulation, time-dependent charge trapping was modeled using Silvaco’s INTTRAP and INTERFACE models, which support transient simulation of interface trap occupation and release during voltage sweeps.

Specifically, we performed quasi-static C–V simulations using forward and backward gate voltage sweeps over the range of –3 V to +3 V. The observed hysteresis arises from asymmetric trapping and de-trapping kinetics at the semiconductor–dielectric interface (particularly in the Al₂O₃ case). These effects were captured by assigning energy-distributed trap states with finite capture and emission times, enabling the simulator to resolve transient charge storage during the voltage sweep.

We have clarified this modeling approach in the revised manuscript under the section discussing hysteresis and C–V characteristics.

**Author action:** We have revised the manuscript

Specifically, we employed the INTERFACE and INTTRAP models to simulate the dynamic trapping and de-trapping of charge carriers during gate voltage sweeps. These models allow for energy-distributed traps with finite capture and emission times, enabling the simulation to account for asymmetrical trap kinetics during forward and backward voltage sweeps. This approach accurately reproduces the observed hysteresis in the Al₂O₃-based device.

-------------------------------------------------END------------------------------------------------------
